# Supplementary material for: Effectiveness of integrated care for older adults with depression and hypertension in rural China: A cluster randomized controlled trial
Source: PLoS Med. 2022 Oct 24;19(10):e1004019. doi: 10.1371/journal.pmed.1004019 (PMC9639850; doi:10.1371/journal.pmed.1004019)
Supplement: S1 Table — (DOCX) [file pmed.1004019.s001.docx]

| **S1 TABLE: Roles and Responsibilities of COACH Team Members** | | |
| --- | --- | --- |
| **Village Doctor (PCP)** | **Aging Worker (AW)** | **Psychiatrist** |
| ***Case Identification and Tracking*** | | |
| Baseline and regular f/u depression screening (PHQ-9) and BP checks, at least monthly. | Standardized baseline in-home assessment of social supports, functional status, medication use, lifestyle, nutrition and financial strain. | Baseline diagnosis (visit to village). |
| ***Depression/HTN Management*** | | |
| **Follow dep treatment guidelines**   - Treatment algorithms. - Systematic follow up. - Ongoing symptom monitoring. - Pt/family education re: adherence, social engagement. - Consult with Psychiatrist as indicated. | **Reduce social barriers to effective Dep/HTN management**   - Monthly visits to assess coping and functioning. - Mobilize community resources to meet basic needs. - Engage and support the family. - Improve communications between patient and PCP. - Decrease social isolation. - Increase nutritional resources. - Support coping with life changes. | Initiate antidepressant as indicated.  Consultation by telephone with PCP monthly and as needed. |
| **Follow HTN treatment guidelines**   - Treatment algorithms. - Systematic follow up. - Ongoing BP monitoring. - Pt/family education re: adherence, diet, exercise.   Consult/refer as indicated with township health center. | **Support adherence to depression and HTN treatment recommendations**   - Increase patient and family’s understanding of the diseases and medications. - Help those unable to afford to obtain medications - Support lifestyle change (e.g., exercise, diet and smoking cessation). - Reinforce medication schedule and adherence (e.g., use of pillbox). - Increase social support and enjoyable activities. |  |
| ***Health education*** | | |
| Teach village older adults and families about mental, cardiovascular health. | Organize village-based education and social events for older adult residents and their families. |  |
| ***Care Coordination*** | | |
| Weekly team meeting re: all COACH subjects – review progress, identify barriers, revise care plan. | | Monthly attendance at team meetings by phone. |
