# Supplementary material for: Effectiveness of integrated care for older adults with depression and hypertension in rural China: A cluster randomized controlled trial
Source: PLoS Med. 2022 Oct 24;19(10):e1004019. doi: 10.1371/journal.pmed.1004019 (PMC9639850; doi:10.1371/journal.pmed.1004019)
Supplement: S2 Table — (DOCX) [file pmed.1004019.s002.docx]

| Outcome | COACH | | eCAU | |
| --- | --- | --- | --- | --- |
| Systolic blood pressure | Mean | SD | Mean | SD |
| Baseline | 144.03 | 14.13 | 145.67 | 13.53 |
| 1 Month | 138.61 | 13.05 | 139.81 | 12.02 |
| 3 Months | 133.69 | 13.38 | 138.59 | 11.62 |
| 6 Months | 132.44 | 12.88 | 137.94 | 11.28 |
| 9 Months | 128.14 | 12.80 | 137.41 | 10.83 |
| 12 Months | 129.48 | 13.15 | 137.09 | 10.32 |
| Diastolic blood pressure |  |  |  |  |
| Baseline | 84.18 | 8.94 | 83.74 | 8.93 |
| 1 Month | 80.47 | 7.76 | 80.60 | 7.64 |
| 3 Months | 79.30 | 7.73 | 80.20 | 7.29 |
| 6 Months | 78.09 | 7.78 | 79.84 | 7.25 |
| 9 Months | 74.31 | 8.37 | 79.24 | 7.17 |
| 12 Months | 75.33 | 8.87 | 79.61 | 7.02 |

**S2 Table: Systolic and diastolic blood pressures for COACH and eCAU participants at each assessment point**
