# Supplementary material for: Effectiveness of integrated care for older adults with depression and hypertension in rural China: A cluster randomized controlled trial
Source: PLoS Med. 2022 Oct 24;19(10):e1004019. doi: 10.1371/journal.pmed.1004019 (PMC9639850; doi:10.1371/journal.pmed.1004019)
Supplement: S3 Table — (DOCX) [file pmed.1004019.s003.docx]

| **S3 Table: Expected/observed care management activities by COACH team members** | | | | | |
| --- | --- | --- | --- | --- | --- |
| Group | PCP | AW | | Team work | |
|  | Patient visits | Patient visits | Community activities organized | Meetings of PCP and AW | Meetings of PCP, AW, Psychiatrist |
| COACH | 12/11.6 | 12/8.3 | 12/9.75 | 48/56.6 | 12/7.9 |
| eCAU | 12/11.3 | n/a | 4/- | n/a | n/a |
| *Notes:* x/y, x = number of expected activities over 12 months of study participation; y = average number actually conducted over 12 months; COACH, Chinese Older Adult Collaborations in Health; eCAU, enhanced care as usual; PCP, primary care provider; AW, aging worker. | | | | | |
