# Supplementary material for: Effectiveness of integrated care for older adults with depression and hypertension in rural China: A cluster randomized controlled trial
Source: PLoS Med. 2022 Oct 24;19(10):e1004019. doi: 10.1371/journal.pmed.1004019 (PMC9639850; doi:10.1371/journal.pmed.1004019)
Supplement: S4 Table — (DOCX) [file pmed.1004019.s004.docx]

**S4 Table**: Depressive symptom severity of study participants over 12 months -- eCAU, COACH who accepted antidepressant medications (Antidep[+]); COACH who declined antidepressant medications (Antidep[-])

|  | **eCAU** | **COACH Antidep[+]** | **COACH Antidep[-]** |
| --- | --- | --- | --- |
| Baseline | 21.76 (3.58) | 22.58 (4.8) | 21.36 (3.98) |
| 3 months | 19.51 (4.87) | 17.22 (5.9) | 19.05 (5.33) |
| 6 months | 19.58 (4.64) | 14.9 (5.14) | 16.72 (4.65) |
| 9 months | 18.85 (4.61) | 13.02 (4.8) | 14.96 (4.31) |
| 12 months | 18.77 (4.67) | 10.7 (3.61) | 15.64 (3.19) |

Note. Depressive symptoms were assessed by the 17-item Hamilton Depressive Rating Scale (HDRS). Figures presented are the mean and (standard deviation) of the HDRS for each group at each time point.
