# Supplementary material for: Effectiveness of integrated care for older adults with depression and hypertension in rural China: A cluster randomized controlled trial
Source: PLoS Med. 2022 Oct 24;19(10):e1004019. doi: 10.1371/journal.pmed.1004019 (PMC9639850; doi:10.1371/journal.pmed.1004019)
Supplement: S5 Table — (DOCX) [file pmed.1004019.s005.docx]

**S5 Table**: Proportion (%) of study participants who had controlled hypertension over 12 months: -- eCAU; COACH who accepted antidepressant medications (Antidep[+]); COACH who declined antidepressant medications (Antidep[-])

|  | **eCAU** | **COACH Antidep[+]** | **COACH Antidep[-]** |
| --- | --- | --- | --- |
| Baseline | 20.21% | 26.75% | 22.78% |
| 3 months | 37.12% | 58.19% | 54.18% |
| 6 months | 37.48% | 59.33% | 54.93% |
| 9 months | 40.88% | 60.59% | 64.86% |
| 12 months | 40.90% | 71.47% | 71.79% |
