# Supplementary material for: Effectiveness of integrated care for older adults with depression and hypertension in rural China: A cluster randomized controlled trial
Source: PLoS Med. 2022 Oct 24;19(10):e1004019. doi: 10.1371/journal.pmed.1004019 (PMC9639850; doi:10.1371/journal.pmed.1004019)
Supplement: S6 Table — (DOCX) [file pmed.1004019.s006.docx]

**S6 Table: Estimated effect size for depression and HTN outcomes in unadjusted analyses**

|  | Estimate | 95% confidence interval |
| --- | --- | --- |
| **Depressive symptoms - HDRS score** |  |  |
| 3 months | 0.46 | 0.33 - 0.58 |
| 6 months | 0.95 | 0.81 - 1.08 |
| 9 months | 1.15 | 1.02 - 1.27 |
| 12 months | 1.37 | 1.25 - 1.49 |
| **HTN Controlled – odds ratio** |  |  |
| 3 months | 2.47 | 1.87 - 3.26 |
| 6 months | 2.58 | 1.92 - 3.45 |
| 9 months | 2.92 | 2.05 - 4.16 |
| 12 months | 4.46 | 3.23 - 6.16 |
