# Supplementary material for: Effectiveness of integrated care for older adults with depression and hypertension in rural China: A cluster randomized controlled trial
Source: PLoS Med. 2022 Oct 24;19(10):e1004019. doi: 10.1371/journal.pmed.1004019 (PMC9639850; doi:10.1371/journal.pmed.1004019)
Supplement: S1 Appendix — (DOCX) [file pmed.1004019.s007.docx]

**The Depression/Hypertension COACH Study**

**Co-Principal Investigators**: Y. Conwell, MD- University of Rochester Medical Center

S. Chen, MD- Zhejiang University, China

**I. PURPOSE OF THE STUDY AND BACKGROUND**

**Purpose of the study**

Overview: The COACH study is a colloborative effort between the University of Rochester Medical Center (PI: Yeates Conwell, MD) and Zhejiang University, China (PI: Shulin Chen, MD). Research activities including-recruimtent, informed consent, data collection, and data safety and monitoring will take place in China under the direction of Dr. Chen at Zhejiang University. The IRB approval letter from Zhejiang University to conduct these research activities is included in this application. Research activies that will take place at URMC under the direction of Dr. Conwell are limited to data management and data analysis in colaboration with the reseach team in China. The PI seeks approval solely for these aspect of the project.

The Depression/Hypertension (HTN) in Chinese Older Adults - Collaborations in Health (COACH) Study is a randomized controlled trial (RCT) comparing the COACH intervention to Care-as-Usual (CAU) for the treatment of comorbid depression and HTN in Chinese older adult rural village residents. COACH integrates the care provided by the older person’s primary care provider (PCP) with that delivered by an Aging Worker (AW; a lay member of the village’s Aging Association), supervised by a psychiatrist consultant. Based on chronic disease management principles, the PCP is trained to use evidence based practice guidelines for treatment of both HTN and depression, and provided with access to mental health consultation regarding optimal management of the patient’s depression. The AW is trained to conduct a systematic assessment of the older person’s social context to identify and reduce social and environmental barriers to treatment adherence and response. AWs participate with the PCP in developing multi-disciplinary care plans for their shared patients, reinforce treatment adherence and adoption of healthy behaviors, and emphasize activation and engagement of the older person in activities designed to improve their connectedness to others and to the community. Finally, PCP, AW, and Psychiatrist Consultant are trained to collaborate in their shared clients’ care. 160 villages will be randomized to deliver COACH or CAU to eligible subjects who reside there (appproximately 15 per village will meet criteria), or a total of about 2400 subjects. Treatment will continue for one year, with research evaluations at baseline, 3, 6, 9, and 12 months. Utilization data will be obtained from the EMR and administrative records. Specific aims of the study are to determine whether COACH is more effective than CAU in treating depression (Aim 1) and HTN (Aim 2); whether improvements in treatment adherence precede reductions in depression and improvement in blood-pressure (BP) control (Aim 3a), and whether improvements in depression symptoms precede improvements in BP control (Aim 3b); if COACH is associated with greater improvements in health-related quality of life than CAU (Aim 4); and to compare the costs associated with each approach (Aim 5).

Depression and HTN are common, costly, and destructive conditions among the rapidly growing aged population of rural China. Because the COACH model provides additional capacity to existing resources in rural settings, integrates social interventions with the medical model, and is consistent with the cultural context of rural life, it would be both a feasible and scalable approach to management of prevalent comorbid medical and mental health conditions of aging in Low- and Middle-Income Countries (LMICs).

**Section A. SPECIFIC AIMS**

Depression is a major public health problem for older adults in China, just as it is worldwide, affecting almost 5% of community-dwelling Chinese over age 60 [1], and a far higher proportion of those with chronic illnesses [2]. Depression is associated with physical illness and functional impairment, greater utilization and cost of healthcare, and increased suicide and all-cause mortality [3]. Cardiovascular disease (CVD) is also highly prevalent and associated with enormous suffering and disability. Hypertension (HTN) is a major risk factor for cardiovascular morbidity and mortality [4-6] as the underlying cause of two-thirds of all strokes and half of all ischemic heart disease [7]. Prevalence of HTN is highest among older adults; in China the estimated prevalence of HTN is 55% of those over age 60 years [8]. The two disorders commonly coexist. Depression is well recognized as a risk factor for HTN [9-12] and for poor adherence to treatment with antihypertensive medications [13-16]. HTN and its sequelae are risk factors for depression, complicating help-seeking, diagnosis, treatment and prognosis [17, 18]. The toll is high. The World Health Organization (WHO) projects that depression and CVD will be the two leading casus of disability worldwide by 2020 [19].

This proposal in response to RFA-MH-13-040 grows from established collaborations in late life mental health research between investigators at the University of Rochester, University of Michigan, and University of Pennsylvania and at major academic institutions (Zhejiang University) and government ministries in China (Zhejiang Provincial Center for Disease Control and Prevention [CDC] and Zhejiang Provincial Committee on Aging [CoA]). It builds on a strong body of preliminary findings and represents the best next step in our shared vision for the development of integrated mental health and primary care service delivery for the enormous, rapidly growing, and still underserved population of older adults in rural Chinese villages.

With the long term **goal** of improving the health of rural Chinese older adults with comorbid mental and chronic physical disorders, our **principal objective** with the Dep/HTN COACH Study is to demonstrate the effectiveness of an integrated care model for treatment of older adults residents of rural Chinese villages in Zhejiang Province who have both depression and HTN. The model we will test addresses both the medical/psychiatric and social/contextual dimensions of illness by integrating two service systems that, although they are already present in every rural village, do not currently provide systematic management or collaborate in care: village doctors (primary care providers [PCPs] administered by the CDC) and the Village Aging Associations [VAAs] administered by the CoA. The model is called **Depression/HTN in Chinese Older Adults – Collaborations for Health** (COACH).

We will conduct a randomized controlled trial in which approximately 2400 residents of 160 rural villages over age 60 with both depression and HTN receive either the COACH intervention or care-as-usual (CAU). Our **specific aims** with this comparison are to compare CAU with COACH in each of the following domains:

**Aim 1 – Depression:** We hypothesize that relative to subjects who receive CAU, those in the COACH intervention will show (Aim 1a) better adherence to antidepressant treatment recommendations and (Aim 1b) greater improvements in their depressive disorders over 12 months of involvement in the study.

**Aim 2 – Hypertension:** We hypothesize that relative to subjects who receive CAU, those in the COACH intervention will show (Aim 2a) better adherence to antihypertensive treatment recommendations and (Aim 2b) greater improvements in blood pressure (BP) control.

**Aim 3 – Temporal associations:** We will examine the temporal associations of change in depression and BP control, hypothesizing that (Aim 3a) improvements in treatment adherence precede symptomatic improvement in depression and HTN, (Aim 3b) improvements in depression will precede improvements in BP control, and (Aim 3c) those temporal patterns are associated with the intervention received.

**Aim 4 –Quality of Life:** In addition to its impact on depression and BP control, we will ask whether the COACH intervention results in greater improvements in health-related quality of life than CAU.

**Aim 5 – Cost:** Finally, further interest in developing the COACH model by the Zhejiang government will hinge not only on its effectiveness relative to CAU, but its costs as well. Therefore, (Aim 5a) we will determine resource utilization and costs associated with delivering the CAU and the COACH interventions over 12 months. We will also(Aim 5b) conduct an exploratory economic feasibility evaluation to guide future implementation and future dissemination research. We anticipate that the COACH model is most effective if used long-term when the impact of reduced depression and BP on a range of health outcomes is fully realized.

Because depression and HTN are such common, costly, and destructive conditions among the rapidly growing aged population of rural China, the COACH model can make a significant impact on older adult health. Because it provides additional capacity to existing resources in rural settings, integrates social interventions with the medical model, and is consistent with the cultural context of rural life, the COACH model would be both a feasible and scalable approach to management of prevalent comorbid medical and mental health conditions of aging in Low- and Middle-Income Countries (LMICs).

**Background and significance**

1. **Background:** This study is in response to RFA-MH-13-040, the purpose of which is to support the study of innovative approaches to integrated care of people in low- and middle-income countries (LMICs) who have both mental illness and one or more chronic medical conditions. Mental illnesses account for a large proportion of the global burden of disease [20]. They commonly co-occur with chronic medical illnesses and interact in complex ways to increase the severity, impede treatment, and worsen the outcomes of both disorders. Approaches to integrating the healthcare of co-occurring mental and physical disorders in high-income countries have shown great promise to improve outcomes [21], but their effectiveness has rarely been studied in LMICs. We will examine *the effectiveness, compared to CAU, of an intervention for co-occurring depression and HTN among adults aged ≥ 60 years receiving care from village doctors (primary care providers [PCPs]) in rural China*. The *“Chinese Older Adult –Collaborations in Health” (COACH) intervention*, proposes to improve both depression and HTN outcomes by utilizing chronic disease management principles and integrating primary care with two additional resource sets -- a county-level psychiatric consultant and the services of a village-resident Aging Worker (AW) to address psychiatric, social and contextual dimensions of depression and HTN care.
2. **Why a focus on depression in older adults?** The World Health Organization (WHO) estimates that by 2020 major depression will be the second leading contributor to global burden of disease measured as disability adjusted life years[19].In China the prevalence of clinically significant mood disorders in later life is significant. Almost 6% of community-dwelling Chinese over age 60 have a depressive illness [1, 22], and among those Chinese with chronic physical illnesses associated with aging, the prevalence of depression is far higher. In Shanghai, for example, over 78% of medical inpatients with HTN were found to have clinically significant depressive symptoms [2]. As well, depression is associated with physical illness and functional impairment, greater utilization and cost of healthcare, and increased suicide and all-cause mortality [3]. Despite its impact, the rate of detection and effective management of depression in China is very low [2, 23]. New approaches are needed to address the burden of depressive illness.
3. **Why a focus on HTN?** HTN is among the most common of chronic medial illnesses, including in China where an estimated 57% of rural village residents over age 60 years are affected [8]. A major risk factor for cardiovascular morbidity and mortality [4-7], HTN is the major underlying cause of *two-thirds of all strokes* and *half of all ischemic heart disease*[7], which is the only disorder to top depression in the WHO’s list of greatest expected causes of global illness burden by 2020 [19].

Like depression, HTN is very poorly recognized and inadequately treated in China. In one recent study of rural Chinese adults aged ≥65 years, only 45% of cases of HTN were recognized. Of further concern, while over 94% of patients who were aware of having HTN were being treated, *BP was controlled in only 5*% [8]. Studies estimate that approximately 20% of people with HTN also have clinically significant depression, which has been associated with the onset and worsening of HTN [9-12], and complicates its management by promoting non-adherence to treatment, further impairing functioning and self-care [16]. The combination of HTN and depression, therefore, as the first and second leading causes of disability adjusted life years by 2020 [19], requires special attention to the development of effective preventive intervention strategies.

1. **Why target rural villages in China?** China is the most populous country in the world, with over 1.34 billion people. While the pace of development is rapid in urban areas, rural regions (where over half of Chinese live) lag behind[24]. Although Zhejiang Province is in a relatively wealthy part of the country, it too faces enormous challenges – almost 70% of its 47.5 million residents live in rural areas and almost 20% of village residents are over age 60 (the true proportion is likely far higher when one takes into account younger and middle-age adults who have moved to the cities to find work yet remain registered as village residents) [25]. The Zhejiang government is receptive to supporting health service system design change, and has been engaged in the design of this proposal. As long as the proposed changes are consistent with the sociocultural context of rural China, they will be given serious consideration for further dissemination.

Helping to drive the Chinese government’s investment in reform is the anticipated rapid absolute and relative growth of the older adult population resulting from increased life expectancy and over 40 years of the “one child” policy. By 2050 over 440 million Chinese will be over age 60 [26] and constitute up to 40% of the population in certain regions (e.g., Makao SAR) [26], even as the dependency ratio (the population aged 65 or over divided by the population aged 15 to 64) rises. Anticipating the impact of these changes on health care costs, the Chinese government requires significant changes in its service delivery models. Recent policy reforms underscore their interest in shifting primary responsibility for chronic disease management to the village PCP [27]. However, with few exceptions [28], innovations in service system design and delivery have not been tested.

**Section C. INNOVATION**

The innovations of the COACH model of care derive primarily from three characteristics: its basis on the collaborative care approach to chronic disease management, emphasis on improving adherence to treatment recommendations, and use of indigenous “aging workers” to address the social and cultural dimensions of care. We describe each here, including relevant preliminary research generated by the COACH Study team.

1. **Collaborative care management:** Disease management approaches that utilize primary care-based case management have been found to improve outcomes associated with a variety of chronic conditions such as cardiovascular disease [29] and depression [30]in various populations including older adults [31, 32]. Few studies, however, have addressed more complex patients--those with comorbid medical and psychiatric conditions.[33]. Recent proposals to incorporate psychosocial with medical and psychiatric dimensions of care hold promise to improve outcomes [34], but have been conducted only in western industrialized nations; none has tested a primary-care based depression care management approach in China.

The COACH model builds on our studies of primary care and community-based approaches to the detection and management of late life depression in China supported by grants to Dr. Chen from both the Department of Science and Technology of Hangzhou (20080333Q14) and the NIHFogarty International Center(R01TW008699). One of these studies[35]focused on the prevalence of late-life depression and its comorbidity with cardiovascular disease (CVD) and diabetes in two urban primary care clinics in Hangzhou. We screened 1275 older adults and interviewed 262 of them with the Structured Clinical Interview for DSM-IV [36]. Results showed a point prevalence of 11.3% for major depression, and that among patients with CVD or/and diabetes the prevalence rates of MDD were 54.6% and 49.2% respectively. On reassessment 12 months later we found that more than *60% of patients with MDD at baseline remained depressed* and that *only three patients (1.1%) had been treated with an antidepressant or referred for specialty mental health care*.

A second study designed to address this disparity formed the basis for the COACH intervention proposed here. Dr. Chen’s ongoing RCT randomly assigned 16 urban Hangzhou primary care clinics to deliver either a collaborative depression care management (DCM) intervention or CAU to patients aged ≥ 60 with major depression [28].The DCM intervention includes use by PCPs of depression practice guidelines, primary care nurses as care managers, and psychiatrists to provide consultation and supervision. Of 242 subjects recruited into the DCM arm of the study,162 (67%) agreed to take antidepressant medications; 164 subjects received CAU. Preliminary results presented in Figure 1 demonstrate the effectiveness of the DCM model.

In combination, these studies show that we can recruit PCPs and older adult subjects with depression from urban clinics in China, so should have similar success in the rural area. They demonstrate the prevalence of depression, including among older patients with CVD in these settings, serving as a guide to our estimates for recruitment in the village clinics. They show that depression is rarely treated, even in an urban clinic where the level of training of the doctors is better than in the rural villages, reinforcing the need for the intervention we have proposed in this study. Also, these results show that almost a third of those who consented did not agree to take an antidepressant. This finding led to our conclusion that the COACH model should emphasize adherence to treatment recommendations and include a social intervention as a supplement or alternative to medications.

1. **Emphasis on Adherence:** Adherence is defined as “the extent to which a person’s behavior (in terms of taking medications, following diets, or executing lifestyle changes) coincides with medical or health advice” [37]. Non-adherence among older adults results in increased hospitalizations [38, 39], health care costs [40], and mortality [41]. Epidemiological studies of western samples have confirmed that on average one in three patients does not complete depression treatment [42-44]. In another study that serves as preliminary data for the COACH intervention, Dr. Bogner demonstrated in analyses of the PROSPECT study data that even under rigorous study conditions less than 40% of elderly primary care depressives were adherent to antidepressant treatment based on pill counts [45]. Adherence to antihypertensive medications is similarly poor [46]. Factors associated with treatment non-adherence include physical limitations [47], cognitive impairment [48], social support [49-51], and the cost and side effects of medications [52-54]. At the provider level, failure to educate the patient about the need for adherence is another important factor [55-57].

Dr. Bogner is currently conducting a treatment development study (R34 MH085880) designed to test the effectiveness of an intervention integrating depression treatment into care for hypertension using a collaborative care model with older adults. Improving adherence to treatment is a central component of the design, informed by a conceptual framework adapted from Cooper and colleagues [58]. It is the only study of which we are aware that examines the impact of adherence support on late life depression care. Early analyses show that patients in the integrated care intervention had lower diastolic blood pressure (intervention 74.2 mmHg vs. usual care 82.0 mmHg; p=.035), and fewer depressive symptoms compared with patients in the usual care group (PHQ-9 mean scores, intervention 2.4 vs. usual care 7.1; p<.001) at 12 weeks, after adjustment for baseline values (personal communication; manuscript pending submission).

No study has yet examined interventions to modify adherence to depression or hypertension treatment in LMICs. The COACH Study will contribute to understanding the role that adherence plays in chronic disease management through its measurement by both self-report and objective approaches (pill counts, prescription records), examining factors shown in the West to be associated with non-adherence, and incorporating adherence support in the COACH intervention (AW support of patients' adherence to medications and lifestyle changes as recommended by the PCP).

1. **Incorporation of Aging Workers (AWs) into the COACH team:** The integration of village AWs into the COACH intervention is an important innovation for three reasons. First, the system of village aging associations staffed by AWs is already in place throughout Zhejiang Province, and in similar forms throughout China. AWs, while not trained as social workers (the discipline of social work is very new in China), serve the same function, albeit in a less structured manner. Standardizing their practice and linking them in a systematic way to the PCP in the service of improving the health outcomes of older adults can be accomplished *without additional expense beyond training*, and is entirely consistent with the cultural mores and expectations of the rural population. Utilization of this existing resource will make dissemination of the model much more feasible.

Second, social stressors are well established risk factors for major depression onset and poor treatment response. Our own research has helped establish the independent role that social factors (impaired social support, social/environmental stressors) play in determining risk for suicide in older adults[59-62], including in China [63-65]. A recent meta-analysis of studies examining the associations between social relationships and non-suicide mortality found that the protective effect of strong social networks was comparable to that of smoking cessation and cardiac rehabilitation among those with cardiac disease [66]. Furthermore, interventions that include strategies to increase social support and access to community services have been associated with reduced risk for suicide, including in older adults [67-70]. With support of an NIMH-funded IP-RISP grant (R24 MH071604; Y. Conwell, PI) Dr. Conwell and collaborators in Rochester have gained extensive experience in the study of the mental illness in older adult clients of community-based aging services providers, and the potential for integrating social services with primary and mental health care providers in a more comprehensive collaborative care model [71-73]. We are not aware of any studies in which the indigenous social service infrastructure in China has been examined for its contributions to older adult health. AWs are well situated to address these same factors as de facto social workers for the village, a role that they are culturally and officially sanctioned to perform. Ours will be the first study to examine their impact.

Other innovative aspects of the COACH Study warrant brief mention. They include the insights it should provide into the mechanism of action of the COACH intervention. We can establish, for example, whether improved adherence mediates the relationship between the intervention and reduced depression and improved BP control, as anticipated by the model that guides our work [58], or whether improvement in depression is necessary for improved BP control among older adults with comorbid illness. Also we will determine the costs as well as the benefits of the intervention, using them in cost-effectiveness (CEA) and cost-benefit analyses (CBA) to provide a basis for future dissemination research on the model. Ours is the first application of comparative effectiveness research to a collaborative care intervention in China of which we are aware.

**II. CHARACTERISTICS OF THE RESEARCH POPULATION**

- 1. **Older Adult Research Subjects:**

**Number of subjects:** A total of 2,400 subjects will be entered into the study.

**Gender and Racial/Ethnic Origin of Subjects:** Subjects will be both male and female. All subjects will be Asian.

**Inclusion Criteria:**  Subjects for inclusion will be: (1) Community-dwelling residents registered to the selected village, and thus also registered patients of the village’s PCP. We will not recruit older adults from hospitals or long-term care settings or temporary residents (i.e., migrants whose residence registration is in another district), because they are not eligible for routine care by village PCPs. (2) Age ≥ 60 years, the typical retirement age in rural China. (3) Clinically significant depression defined as baseline PHQ-9 score ≥ 10. (4) Systolic BP ≥ 140 or diastolic BP ≥ 90 for non-diabetics, or a systolic BP ≥ 130 or diastolic BP ≥ 80 for patients with DM (standards defined by the Joint National Committee on Prevention, Detection, and Treatment of High BP [1]. (5) Intact cognitive functioning (6-Item Screener score <3) to assure ability to participate with the treatment team in management of their conditions. (6) Capable of independent communication, which is necessary both for valid research assessment and for participation in the intervention. Hearing or vision problems are very common in older people. If subjects have vision problems but can hear and understand the conversation in person, they will be included. If a subject has mild or moderate hearing problems but can communicate with hearing aids, they will also be included. (7) Willing to give informed consent. We will test their capacity to consent by questioning their understanding of the consent form as presented by the research assistant prior to enrollment.

**Exclusion Criteria**: (1) Incapable of giving verbal consent to this study. Patients who are unable to comprehend the purpose and procedures of the study, appreciate the risks and alternative, or agree to the study for irrational reasons will be considered unable to provide consent. We will test their capacity to consent by questioning their understanding of the consent form as presented by the research assistant prior to enrollment. (2) Acute high suicide risk at baseline assessment. If patients are found to be at high suicide risk based on the RA’s assessment at intake, they hey will be excluded from the study at that time and referred to the PCP for further assessment, with backup available from the County Mental Hospital psychiatrist. Patients assessed to be dangerously suicidal at later assessments will be discontinued from the study, their providers notified, and their safety guaranteed. (3) Psychosis, alcoholism. We exclude patients with psychosis or active alcoholism in the past 6 months. Few older adults (3.4%) have heavy alcohol drinking in rural China; and the current rate of alcohol abuse in elderly is lower than 1% [2].

**Vulnerable Subjects**: Older adults are targeted for this study because the purpose of the study is to explore depression/ hypertension specifically in adults age ≥ 60 years. As mentioned above the stringent inclusion/exclusion criteria protects those older individuals considered to be more vulnerable.

**1.2 Primary Care Providers (PCPs) and Aging Workers (AWs):**

**Number of subjects**: 160 villages will be selected for inclusion in the study and randomized to deliver either the COACH intervention or care-as-usual (CAU) to its residents who meet criteria and consent to participate. Each village has one PCP and an aging committee, of which one member is the identified AW for the village. In the CAU arm of the study, only the village PCP will participate as a research subject, because CAU does not include AW involvement. In COACH villages, both the PCP and AW will be invited to participate. If both PCP and AW do not agree in villages designated to the COACH arm, that village will no longer participate and another will be chosen from the 283 villages in Tonglu County, the site of the study.

**Age, Gender, and Racial Origin of PCP Subjects**: PCPs are typically between the ages of 24 and 60 years, with an approximately equal distribution of men and women. All are Han Chinese.

**Inclusion/Exclusion Criteria for PCPs:** All are eligible to participate as long as they are actively employed as a study village’s PCP by the Zhejiang Provincial Center for Disease Control (CDC) and are willing to provide informed consent.

**Age, Gender, and Racial Origin of AW Subjects**: AWs are village residents between the ages of 40 and 70 years, approximately equal proportions of men and women, who are paid part-time with responsibility for engagement and education of the village’s older adults. All are Han Chinese. They typically are fully literate, with about 8 years of schooling, and well respected by others, s/he knows all older adults in the village and their social circumstances.

**Inclusion/ Exclusion Criteria**: All are eligible to participate as long as they are identified as a study village’s AW by the Zhejiang Provincial Committee on Aging (CoA) and are willing to provide informed consent.

**III. METHODS AND PROCEDURES**

**Methods and Procedures:**

**1. Overview:** The Dep/HTN COACH Study is a randomized controlled trial (RCT) comparing the COACH intervention to CAU for the treatment of comorbid depression and HTN in Chinese older adult rural village residents. COACH integrates the care provided by the older person’s PCP with that delivered by a lay Aging Worker (AW) from the village’s Aging Association, supervised by a psychiatrist consultant.

Based on chronic disease management principles, the **PCP** is trained to use evidence based practice guidelines for treatment of both HTN and depression, with access to mental health consultation regarding optimal management of the patient’s depression. The **AW** is trained to conduct a systematic assessment of the older person’s social context to identify and reduce social barriers to treatment adherence and response. AWs participate with the PCP in developing multi-disciplinary care plans for their **shared patients**, reinforce treatment adherence and adoption of healthy behaviors, and emphasize activation and engagement of the older person in activities designed to improve their connectedness to others and to the community. Finally, **PCP, AW**, and **Psychiatrist Consultant** will be trained to collaborate in their shared clients’ care.

We seek to determine whether COACH is more effective than CAU in treating depression (**Aim 1**) and HTN (**Aim 2**); whether improvements in treatment adherence precede reductions in depression and BP (**Aim3a**) and whether improvements in depression symptoms precede improvements in BP control (**Aim3b**); if COACH is associated with greater improvements in health-related quality of life than CAU (**Aim 4**); and to compare the costs associated with each approach (**Aim 5**).

The villages, each of which has only one PCP, will be the unit of randomization. 160 villages will be randomly selected from Tonglu County in Zhejiang Province, and then randomly assigned to CAU or COACH. Villages will be selected in a manner assuring that no two are geographically contiguous. Older adult residents from the selected villages who have both depression and HTN will then be recruited into the study. After giving informed consent, they will undergo a baseline research evaluation; receive treatment for 12 months with the approach to which their village was assigned; and be re-evaluated at 3, 6, 9, and 12 months after entry. Costs of care received during the intervention will be calculated from electronic health records.

1. **Setting** – **Rural villages of Tonglu County in Zhejiang Province, China**

The study will be conducted in rural villages of Tonglu County, one of 55 counties in Zhejiang Province. Zhejiang is a province in eastern China with a total population of 47.5 million people, of whom 32.8 million (69.1%) are rural residents. Its capital is Hangzhou.

We selected **Zhejiang Province** because, as the home of our Chinese collaborators, it is easily accessible for study implementation and oversight, and because provincial government officials have shown enthusiastic support for this study and for our previous work in urban Hangzhou primary care clinics [28, 35, 74]. The ability to compare results of this study with those of ongoing work comparing collaborative care management for late life depression with CAU in urban Hangzhou is of added value. As well, even though its rural areas are relatively impoverished, Zhejiang is a progressive, rapidly developing province that competes to set a standard for modernization in China. Findings concerning effectiveness of the COACH intervention will have significant influence on policy and implementation of health services in Zhejiang, but in other regions also, based on the Zhejiang experience, as their health and human services infrastructures develop.

We elected to recruit from one representative county within Zhejiang rather than from numerous counties in order to make most efficient use of the study resources. Villages within one county are all within manageable traveling distance of the County’s mental hospital and township health centers. While there is some variability between counties, for example, in mean household income, the variability is small. We selected **Tonglu County** as the site for the study because its *size and average household income are both at the mean of all counties in the province*. Tonglu County encompasses 283 villages with an average population of 1900, of which approximately 16.8% are age ≥ 60. They are served by a total of 283 PCPs (one in each village). There are 32 Township Health Centers to provide backup to the village PCPs for medical care. But like other rural counties in Zhejiang, Tonglu County has only one Mental Hospital. It houses 8 psychiatrists to serve the mental health care needs of all 0.65 million people, of which 0.52 million are registered village residents. Every village has an Aging Association (VAA) to serve and promote the wellbeing of its elderly residents. The VAA is typically staffed by five people, *one of whom is the designated AW* (ordinarily the education officer). Therefore, *each village has one designated PCP and one AW available to constitute* ***the collaborative care team*** *in those villages assigned to deliver the COACH intervention with consultation from one of 8 County Psychiatrists*.

1. **Randomization strategy and procedures:** The unit of randomization will be the village. Villages are small (average 1900 residents) *with all residents cared for by one PCP and one VAA*. Village randomization is equivalent to PCP randomization. There is much sharing of information within the village; indeed, this is the basis for the hypothesized impact of the AWs’ social connectedness functions in the COACH model. Randomization at the patient level would result in cross-contamination of treatment arms within a village.

All 283 villages in Tonglu County will be eligible for randomization, of which 160 will be selected and their PCPs and VAAs assigned to deliver the CAU or COACH models of care. Villages will be selected in four “waves” (see Figure 2) in order to spread recruitment and training out over four years.

As an additional guard against contamination of one intervention by the other, we will assign villages to treatment arms so that no two delivering different interventions are immediately contiguous. It will also be important that PCPs (and AWs in villages whose PCPs are assigned to the COACH intervention) are *willing to participate* in the treatment arm to which they are assigned. Therefore, upon random selection of the village in which they work, each PCP and AW will be approached by the study coordinator and their County-level supervisors for agreement to participate. Their verbal informed consent will be obtained. If any PCP or AW declines, or if their assignments place them immediately adjacent to a village in which the other intervention is to be offered, another village will be selected from the pool. Given the large number of villages to choose from and the interest of our government agency partners in the study, we expect no problem identifying and engaging sufficient PCPs and AWs.

| **Figure 2** | | Villages randomized | Subjects entered | **Year 1** | | | | | | **Year 2** | | | | **Year 3** | | | | | **Year 4** | | | | | **Year 5** | | | |
| --- | --- | --- | --- | --- | --- | --- | --- | --- | --- | --- | --- | --- | --- | --- | --- | --- | --- | --- | --- | --- | --- | --- | --- | --- | --- | --- | --- |
|  |  |  |  | 1 | 2 | | 3 | | 4 | 1 | 2 | 3 | 4 | 1 | 2 | 3 | 4 | | 1 | | 2 | 3 | 4 | 1 | 2 | 3 | 4 |
| Set up | |  |  |  |  | |  | |  |  |  |  |  |  |  |  |  | |  | |  |  |  |  |  |  |  |
| Wave 1 | | 40 | 600 |  |  | |  | |  |  |  |  |  |  |  |  |  | |  | |  |  |  |  |  |  |  |
| 2 | | 40 | 600 |  |  | |  | |  |  |  |  |  |  |  |  |  | |  | |  |  |  |  |  |  |  |
| 3 | | 40 | 600 |  |  | |  | |  |  |  |  |  |  |  |  |  | |  | |  |  |  |  |  |  |  |
| 4 | | 40 | 600 |  |  | |  | |  |  |  |  |  |  |  |  |  | |  | |  |  |  |  |  |  |  |
| Site visits by oversight committee | | | | | | | x | | x | x | x | x | x | x | x | x | x | | x | | x | x | x |  |  |  |  |
| Analysis | |  |  |  |  | |  | |  |  |  |  |  |  |  |  |  | |  | |  |  |  |  |  |  |  |
|  | Study prep and analysis | | | | |  | | PCP/AW training | | | | | | | | | |  | | = Intervention | | | | | | | |

1. **Human Subjects Involvement and Characteristics:** Two groups will be included as research subjects: 1) older adults with comorbid depression and hypertension; and 2) the primary care providers (PCPs) and Aging Workers (AWs) involved in the older adult subjects’ care.
2. **Recruitment and study assignment procedures:** The village is the unit of randomization. One hundred and sixty villages will be randomly selected from Tonglu County in Zhejiang Province as sites from which subjects will be recruited. Each village has one PCP and one Aging Association represented in the study by one Aging Worker (AW). Random selection and assignment of villages and their associated PCPs and AWs will be performed by a remote computer-generated number sequence concealed from researchers that will be developed and administered by Xin Tu, Ph.D. and Dong Hengjing, PhD, our biostatisticians from University of Rochester and Zhejiang University. Eighty villages and their associated PCPs and AWs will be assigned to deliver the Dep/HTN COACH (COACH) intervention and 80 to deliver CAU. In the CAU villages, only the PCP will be recruited as a study participant, because the AW has no role in the intervention in that treatment condition.

Study personnel will contact each village PCP and AW, explain the study, and solicit their cooperation, and obtain their informed consent to participate. Next the PCP in each study village will generate a list of all adults over age 60 in the village with a diagnosis of HTN. The PCP will then obtain verbal informed consent from each of these individuals to administer the PHQ-9, for which they will be trained with regard to administration and interpretation by the RA, to identify those with clinically significant depression (PHQ-9 score ≥ 10). The PCP will then obtain the verbal consent of those who screen positive to release their name and contact information to the study team. The PCP finally will introduce the potential subject to the RA, who will seek informed consent to participate in the study.

Tonglu County data show that the average number of residents in a village is 1900, 16.8% (319) of whom are aged 60 years or more [3]. The prevalence of HTN in rural China is more than 40% [4], of which approximately 5% are receiving treatment that controls their blood pressures (BP). Approximately 20% of HTN patients also have depression [5]. Therefore, there will be on average 24 patients in each village ≥ 60 years old with depression and HTN (PHQ-9 score ≥ 10 and systolic BP > 140 or diastolic BP > 90 or, if the patient has diabetes, then systolic > 130 and diastolic > 80) who are potential subjects for the COACH study. Our experience in recruiting older adult primary care patients with depression into a collaborative care management trial in Hangzhou has been that 70% consent to participate. Therefore, as a conservative estimate, we expect that 15 subjects will be eligible and consent to participate in each village, a total of 2400 subjects (160 villages with 15 subjects each); 1200 residents of COACH intervention villages and 1200 from CAU villages.

1. **Retention strategies:** Subjects will be evaluated at baseline, 3 months, 6 months, 9 months, and 12 months after study entry. There is little migration by older adults in rural China, and because the villages are small and the PCP for the village cares for all its residents, we anticipate no problem tracking subjects from one assessment point to the next. Subjects will be reimbursed for their time, further helping to assure their retention through the full year of study participation.

PCPs and AWs are also stable members of the community. With full support of their CDC or CoA supervisors, PCPs and AWs will integrate their study duties into their regular work flow. However, there will be some additional work involved, for example in maintaining study logs and answering questions about their own experience with the study, for which each will be compensated.

1. **Interventions**
   1. **Care-as-Usual (CAU)**

PCPs in Zhejiang Province are residents of the villages where their clinics are based. They have on average 3 years of medical education after completing high school. They receive systematic training in the diagnosis and treatment of common chronic medical illnesses, and are expected to follow guidelines for management of HTN developed by China CDC [76]. Analogous to guidelines developed by the U.S. Joint National Committee on Prevention, Detection, Evaluation, and Treatment of High Blood Pressure (the “JNC 7 Report”; [4]), they address detection, management by both pharmacologic and non-pharmacologic means, and guidance on when to refer to more specialized care. These guidelines constitute CAU for HTN in this study.

Village PCPs, however, receive little training in the diagnosis or treatment of mental disorders, and they are provided with no practice guidelines to help with its management. When depression is suspected by PCPs, current practice involves suggesting to patients (or family members) that they consult a psychiatrist at the County Mental Hospital for diagnosis and treatment. There is no direct referral/transfer mechanism between PCPs and mental health specialists, and it is uncommon for patients to take the initiative. Provincial laws prohibit the village PCP from initiating antidepressant treatment. However, if the patient does see the County Mental Hospital psychiatrist who then begins antidepressant medication, the PCP may choose to renew the prescription, typically in two-week increments.

PCPs assigned to CAU will be told when study subjects screen positive for depression, and will be provided with copies of the HTN and depression practice guidelines developed by Dr. Chen for the Hangzhou late life depression care management study. CAU physicians will see their patients when they deem it necessary, handling any problems that may arise in the management of their patients’ depression or HTN. Physicians may refer the patients to specialists as indicated.

Informing PCPs of that their patients may be depressed and providing them with copies of the practice guidelines is an enhancement of CAU. However, we do not anticipate that it will substantially impact patient outcomes. Prior studies have shown that screening, identification of depression, or provider education has little effect on patient outcomes without other chronic disease management intervention components [80-84].

- 1. **Depression/HTN in Chinese Older Adults - Collaborations in Health (COACH)**

The COACH intervention applies chronic disease management principles to the treatment of both depression and HTN by the integrated efforts of the village PCP, an AW (a designated member of the Village Aging Association), and a consultant psychiatrist (referred to here as the Psychiatrist.). We will first describe the backgrounds and qualifications of each team member, their roles and responsibilities on the team, procedures guiding their interactions, and the training they will receive to fill their roles.

- - 1. **Background and Qualifications**
       1. **PCP:** The background and qualifications of the PCP are as described above for CAU.
       2. **AW:** Each VAA is ordinarily staffed by 5 people who are village residents (please refer to the Resources section for explanation of Aging Association staffing).One is a paid part-time worker with responsibility for engagement and education of the village’s older adults. Typically more educated (fully literate, with about 8 years of schooling) and well respected by others, s/he knows all older adults in the village and their social circumstances. This person will be the designated AW for the village. Although the AWs have no special training in social work, they receive in-service training from the Bureau of Civil Affairs and have knowledge and experience in conducting the work of the VAA, which includes:
- Being knowledgeable about community resources and facilitate older residents to apply for eligible services and benefits(e.g., health insurance and old age allowance).
- Provide social and educational activities for older residents, including those in support of public health (e.g., promote physical activity and healthy diet). These activities and other VAA functions take place in residents’ homes as well as a village community center.
- Serve as an ombudsperson, together with other VAA volunteers, for elderly residents.
- Address problems older residents may encounter (e.g., financial strains, family discord, social isolation).
  - - 1. **Psychiatrist:** Each COACH team will be linked with one of the eight psychiatrists based at the Tonglu County Mental Hospital. The hospital has a full array of inpatient (40 beds) and outpatient services. The psychiatrists have on average 5 years of medical training followed by 2 years of specialty training. Their scope of practice includes psychiatric diagnosis and management of patients with mental illness from across the county. Their role includes initiating treatment with any psychotropic drugs. Subsequent prescriptions can be written by village PCPs; however, few feel comfortable doing so given their lack of education and training. Consequently, it is rare that mental disorders, including depression, are treated.

| **TABLE 1: Roles and Responsibilities of COACH Team Members** | | |
| --- | --- | --- |
| **Aging Worker (AW)** | **Village Doctor (PCP)** | **Psychiatrist** |
| ***Case Identification and Tracking*** | | |
| Baseline in-home assessment of social supports, functional status, medication use, lifestyle, nutrition and financial strain using a standardized instrument. | Baseline and regular f/u depression screening (PHQ-9) and BP checks. | Baseline diagnosis (visit to village). |
| ***Depression/HTN Management*** | | |
| **Reduce social barriers to effective Dep/HTN management**   - Weekly visits until depression& HTN controlled. - Mobilize community resources to meet basic needs. - Engage and support the family. - Improve communications between patient and PCP. - Decrease social isolation. - Increase nutritional resources. - Support coping with life changes. | **Follow dep treatment guidelines**   - Treatment algorithms. - Systematic follow up. - Ongoing symptom monitoring. - Pt/family education re: adherence, social engagement. - Consult with Psychiatrist as indicated. | Initiate antidepressant as indicated.  Consultation with PCP as needed (telephone). |
| **Support adherence to depression and HTN treatment recommendations**   - Increase patient and family’s understanding of the diseases and medications. - Help those unable to afford to obtain medications - Support lifestyle change (e.g., exercise, diet and smoking cessation). - Reinforce medication schedule and adherence (e.g., use of pillbox). - Increase social support and enjoyable activities. | **Follow HTN treatment guidelines**   - Treatment algorithms. - Systematic follow up. - Ongoing BP monitoring. - Pt/family education re: adherence, diet, exercise. - Consult/refer as indicated with township health center. |  |
| ***Health education*** | | |
| Organize VAA-based education and social events for older adult residents and their families. | Teach village older adults and families about mental, cardiovascular health. | Participate in VAA education activities re: mental health. |
| ***Care Coordination*** | | |
| Weekly team meeting re: all COACH subjects – review progress, identify barriers, revise care plan. | | Monthlyattendance at team meetings by phone. |

- - 1. **Roles and Responsibilities of COACH Team Members:** The domains of responsibility and associated activities for each team member are delineated in Table 1. Consistent with chronic disease management and collaborative care model design [85-88], they include (a) case identification and tracking, (b) use of evidence-based practices applied according to established guidelines, (c) systematic follow up for outcomes monitoring, (d) engagement of the patient/family in their care, (e) a multidisciplinary team of providers, and (f) access to consultants for advice on management of more complex patients.

It is important to note that whereas most collaborative depression care management models utilize a nurse and/or depression care specialist embedded in the primary care practice, rural Chinese village clinics ordinarily are staffed only by the PCP. There is no nurse. *We have chosen to link the PCP to the AW rather than hiring nurses for these clinics for several reasons*. To add a nurse to each clinic would be expensive and unlikely to be sustained. It would be difficult to find sufficient nurses in China to fill those positions. AWs are now in place in all rural villages, a well-established and accepted resource that to this point has not yet been linked systematically with the village PCP to address the common health problems of older adults. And most importantly, social context is an important determinant of health and illness. Engaging the AW to help reduce barriers to effective disease management and support treatment will yield better outcomes at lower cost.

- - 1. **Procedures Guiding Team Implementation of COACH**

Villages (and their resident PCPs and AWs) will be selected in four waves. In each wave 40 villages will be randomly assigned to either the CAU or COACH condition. The PCPs and AWs of villages assigned to implement COACH will then undergo training in first three months at the Tonglu County Mental Hospital along with the Psychiatrist Consultant. The content of that training will be described in the next section. Thereafter they will deliver the intervention. Older adult subjects will be evaluated at baseline then followed for 12 months. Twelve months of active treatment should be ample time in which to see an impact on the outcomes of interest. We describe here the workflow of intervention implementation:

- As training draws to a close, the PCP for each village will identify all patients over age 60 with HTN diagnoses through review of the electronic medical record. Then, with training in the PHQ-9 by the RA, the PCP will reach out to each potential subject to complete an initial PHQ-9 screen for clinically significant depression and check their BP using research grade methods [4]. Those with PHQ scores ≥ 10 and elevated BP will be invited to meet with the RA after providing verbal informed consent to release their contact information to research staff members.
- Shortly thereafter the RA, who will be based at Tonglu County Mental Hospital, will travel to the village, meet with all potentially eligible subjects who give verbal informed consent to do be contacted, obtain written informed consent from those willing to participate, and conduct the baseline research assessment.

At that point, the subject’s participation in the study will begin. CAU subjects will be managed as previously described. *The following procedures apply to implementation of the* ***COACH intervention only***:

- The PCP will meet with the patient to review management of their BP.
- The Psychiatrist will travel from Tonglu to each COACH intervention village and conduct the diagnostic assessment in the subject’s home, the village clinic, or the VAA, according to the subject’s preference. S/he will then consult with the PCP and, if medications are indicated, initiate treatment with an antidepressant according to the study’s treatment guidelines. (Note that major depression diagnosis is not a requirement for inclusion, and neither is antidepressant prescription.)
- The AW will, within the first week, visit the subject in his or her home to conduct a systematic assessment of the person’s functional status, social supports, lifestyle, medication use, nutritional and financial status using a structured assessment tool based on one we developed in Rochester called the Geriatric Wellness Screen (GWS).
- The AW and PCP will then meet, review the findings of the AWs in-home assessment and the patient’s physical and mental health, and construct a care plan (problem list, approach to each problem, responsible person, target date for completion) that addresses social, physical, and mental health needs in a coordinated fashion.
- The PCP will continue to meet with the patient at the intervals designated by the HTN and depression practice guidelines, monitoring progress with repeat PHQ-9 and BP measures.
- The AW will continue to work with the subject to address identified problems (instrumental assistance); educate the subject and family about their illnesses and support adherence to depression and HTN treatment regimens; encourage good health behaviors (diet, exercise, smoking cessation) based on established psychoeducational methods (e.g., [89, 90], available in Chinese); and attempt to connect the patient to others and to the community (e.g., engage in social groups at the VAA, visits with friends and family). These activities can be done through working with individuals (e.g., home visits with the subject and family), small groups (e.g., cooking demonstrations, Tai Chi class), and community events (e.g., communal meals, games.) Outreach to the subject will continue weekly for 2 months, then biweekly for 2 months, then monthly. If the patient continues to require AW support, more frequent visits will be allowed. (Note that regular home visits within the village to older adults are routine practice for, and expected of, AWs).
- The PCP and AW will meet weekly to review their shared caseload. Each patient will be very briefly discussed and the treatment plan updated as indicated. The Psychiatrist will join these team meetings monthly to make additional suggestions regarding depression management.
- PCP may call the Psychiatrist between the monthly team meetings for additional consultation, and patients may be referred to the County Hospital as needed to assure safety.

As noted below, we expect to recruit approximately 15 older adults subjects from each village, making this level of activity feasible for COACH team members.

- - 1. **Training Requirements and Procedures**

Neither the PCP nor the AW in CAU-assigned villages will receive additional training. However, each member of the COACH team requires training in their individual role, and all require training in how to work together collaboratively. Training for each wave of village providers will be conducted at the Tonglu Mental Hospital education center during the three-month period preceding the 12-month intervention (see Figure 2).

The PCPs and AWs will spend a total of 7 days at the training center – an introductory 5-day session followed two months later, after initiation of the intervention, by a 2-day visit to consolidate their learning. During the introductory session, the first 4 days will be role specific training. On the fifth day the PCPs, AWs, and Psychiatrists will join for one day of combined training on collaborative team functioning.

The **curriculum for the PCPs** includes four major components: (a) **depression management** following the guideline used in Dr. Chen’s GRIP study (urban primary care settings), which adapted the Duke Somatic Treatment Algorithm for Geriatric Depression (STAGED)[91] to just two stages (see Appendix 1 for the adapted Chinese language document). The first stage is 12 weeks of treatment with sertraline or citalopram; the second stage is another 12 weeks of treatment augmentation with bupropion if patients fail to respond in the initial trial. For more complicated cases, transfer to county psychiatrists is indicated. (b) **HTN management** using the guideline adopted by the primary care system in China[76]. All village PCPs have been trained how to use the guideline and receive in-service training yearly to reinforce their adherence to this guideline; COACH training will be review and integration with depression management. (c) **Case management** using the toolkit adapted from the MacArthur Initiative on Depression in Primary Care [92] and being used now in the urban Hangzhou primary care study[28]. We will teach the PCPs to integrate management of depression and HTN using this approach. (d) Finally, **psychoeducation (PE) and communication skills** are also important components. Drs. Li and Chen have developed and translated to Chinese a training manual about depression in later life and communication skills with older Chinese for use in urban Hangzhou. Dr. Chen will deliver the depression related training, and Dr. Yu (HTN specialist) will deliver the HTN related training.

Simultaneously the AWs will convene with Drs. Li and Mao for training in their role. The **AW curriculum** will include: (a) **Overview** of depression, HTN, and their relationship. (b) **Self-management** of depression and HTN. (c) Social environment as **risk and protective factors**. (d) Conducting **psychosocial assessment** and **developing a care plan**. (e) Providing **psychoeducation** to subjects and their families. (f) Techniques to **reinforce treatment adherence** and healthy lifestyles. (g) **Communication** and problem-solving skills with older adults and their families. (e) Ethical standards including **confidentiality**.

For the final day, PCPs, AWs, and Psychiatrists will come together to learn how to collaborate in care management. They will be taught about the roles of the other team members and review procedures guiding their communications and information transfer. Instruction techniques will include structured didactics, role plays, and process/problem solving sessions exploring potential barriers to communication and collaboration.

- - 1. **Fidelity Checks:** Fidelity to the intervention will primarily take the form of standard implementation evaluation. Having provided informed consent to participate as research subjects as well, PCPs, AWs, and Psychiatrists will maintain **logs** of their participation in team meetings and contacts with subjects, their families and other team members. Dr. Mao will review all logs on a monthly basis to assure sufficient contact and coordination of care. Where the observed numbers fall below expectations (e.g., weekly PCP/AW team meetings not attended), Dr. Mao will intervene. As well, we will monitor quarterly select **quality metrics** available from the EMR and insurance database to check PCP adherence to guidelines (e.g., frequency of visits with depressed patients). Direct observation or ratings of recorded interactions is beyond the scope of the study.

1. **Measures**

The measures we will obtain from older adult subjects fall into three domains: socio-demographic characteristics, outcome variables specified by the aims, and covariates that may independently influence adherence and treatment response.

- 1. **Socio-demographic characteristics and baseline clinical assessment**: Information will be collected using a standardized form developed for the study and administered at baseline only (*all other measures will be administered at baseline, 3-, 6-, 9-, and 12-month follow up*). Variables derived will include age, sex, education and literacy level, marital status, and number of children. We will use the Mini-International Neuropsychiatric Interview (MINI) [93], a yes/no instrument that derives DSM-IV-R diagnoses on the basis of respondent's reports of their symptoms to assess for bipolar disorder, psychosis, and alcohol misuse disorder diagnoses, which are exclusion criteria, and to more fully characterize the subject’s psychopathology (note, however, that subject inclusion is based on PHQ-9 score, not categorical diagnosis). If a question of suicide risk is raised, the study’s safety protocol will be implemented and a decision made in that process whether the subject should be excluded from the study. Testing of cognitive capacity is described below.
  2. **Outcome variables**:

**Aims 1a &2a**: We will take two approaches to measurement of **adherence** to antidepressant and antihypertensive medication recommendations – one self-report and the other objective. First, we will use the **Morisky Medication Adherence Measure** (MMAM) [94] to assess the subject’s antidepressant and antihypertensive medication use behaviors. Its 8 items include questions relevant to treatment of both disorders (e.g., “Do you sometimes forget to take your [health concern] pills?” and “When you feel like your [health concern] is under control, do you sometimes stop taking your medicine?”. Sores range from 0 (poor adherence) to 11 (full adherence). The measure has been widely used in HTN adherence studies, including in China, where it has been found to have good reliability and validity [95]. Our second approach will be to construct a **Medication Possession Ratio** (MPR) from a combination of pill counts and verification of pharmacy refills obtained from the clinic’s EMR. The MPR is an accepted metric for the evaluation of adherence using retrospective data, including in China [96]. Prospective data collection, such as by using a Medication Event Monitoring System, is too costly. Patients will be requested at baseline to keep all bottles (including any that are changed) until the follow-up visit. The RA will record from each bottle the date the prescription was dispensed, the number of pills provided by the pharmacy, the dose and frequency of the prescription, and the number of pills remaining in the bottle. Subjects will be asked if they have received oral instructions to change the dose or frequency. If such a change was made without a change in the prescription bottle, the subject’s self-report will be recorded. Also, the billing and dispensing records available from the insurance database will be used to compete the record. The MPR then is calculated as the ratio of the total days of medication supplied to total days in a period [97]. We will calculate the variable for analysis both as a continuous and as a dichotomous outcome, with values ≥80% considered medication adherent [98].

**Aims 1b:** The measure of **depressive symptom change** for Aim 1b will be the **Hamilton Depression Rating Scale** – 17 item version (HDRS), which is widely used, reliable and valid in Chinese [99]. Consistent with other geriatric depression studies [100] and our own work[101], we will examine HDRS change as a continuous variable and define response to treatment as a change in the HDRS scores >50%and remission as a follow up HDRS score lower than 10.

**Aim 2b:** Improvement in BP control will be defined as a **BP reading** at follow up, with normal as specified by the HTN Treatment Guidelines [4, 76]. BP will be taken according to JNC 7 standards (auscultation with cuff deflation method after seated quietly for 5 minutes in the proper position; no caffeine, exercise or smoking in preceding 30 minutes; appropriately sized cuff; two measurements and the average recorded.)

**Aim 3:**Analyses regarding temporal associations will use the variables described above for Aims 1 and 2.

**Aim 4: Quality of life** will be measured using the **WHOQOL-BREF**[102, 103], a 26 item scale that yields four domain scores - physical, psychological, social and environment - each of which we will examine separately in analyses. The WHOQOL-BREF is a widely used measure the Chinese version of which has shown very good psychometric properties [104-106]. As well, we will measure satisfaction with one’s healthcare using the **Client Satisfaction Questionnaire 8-item** (CSQ-8), a well-validated measure in the West that we have adapted and currently use in our Hangzhou study [107, 108].

**Aim 5:** To estimate the costs associated with the intervention, we will evaluate two components: (a) the incremental costs of adding COACH resources to CAU (**programmatic costs**), and (b) **medical costs** attributable to the care of the subjects in each arm. Programmatic costs will include expenses associated with training, travel for consultant psychiatrists between Tonglu and the villages for the intake assessment, staff time for intervention team meetings, and any information system costs. Medical costs include costs of medical treatment documented by the subjects’ EMR and Zhejiang Province insurance data that document each office visit, drug prescription, laboratory test performed, or hospital stay for medical or mental health reasons. As well, we will include patient out-of-pocket healthcare costs in the preceding 3-month interval obtained by subject interview at each research assessment point. Cost data will be converted to US dollars.

- 1. **Covariate measurement** is included to better account for factors known to influence adherence, treatment response or both. They include the following. **Risk factors for cardiovascular disease**: The **Charlson Comorbidity Index** [109],adapted for use with ICD-9 codes [110], supplemented by questions about common disabling conditions of late life including DM, high cholesterol, heart disease, and stroke; and the **Body Mass Index** (BMI), which is associated with HTN treatment response. **Function:** As a measure of functioning independent of the WHOQOL physical domain score, we will measure the subject’s impairment in basic (**ADL**) and instrumental activities of daily living (**IADLs**) [111]. **Cognition:** The **Six-Item Screener** (SIS) [77] is a short standardized mental status examination that has been widely employed for clinical and research purposes for global assessment of cognitive functioning, including in China [112]. **Social Support:** The Chinese version of the **Medical Outcomes Study Social Support Survey** (MOS-SSS-C) is relatively short (20 items), well validated, includes four subscales tapping into important elements of social support (instrumental support, emotional support, positive social interaction, and informational support), and was developed for use in chronically ill samples including Chinese older adults [113]. As a measure of **social network size** we will use the total number of persons with whom the respondent has discussed important matters in the past 6 months, a measure commonly employed in personal social network studies including in China [114]**. Cost of Medications:** The cost to patients of their medications will be obtained from the EMR and insurance claims data. In addition, participants will be asked to rate their overall level of financial burden by their level of agreement to two statements - “I have difficulty paying for health care” and “I have difficulty paying for my medicines.” **Side Effects of Medications:** Participants will be queried about side effects of both their antidepressant and antihypertensive medications using the **Antidepressant Side-Effect Checklist**[115] and the **Side-Effects and Symptoms Distress Checklist** [116] respectively. **Adequacy of antidepressant treatment** will be recorded using the **Composite Antidepressant (CAD) score** developed by Keller [117] and adapted for geriatric patients by Alexopoulos et al. [118]. **Perceived Stigma:** To assess patient’s attitudes regarding medication treatment for depression, we will use three items adapted in our previous studies from Givens et al [119]. They include: If I were taking a prescribed medication for depression: a) I would feel ashamed (yes/no), b) I would feel comfortable telling my friends or family (yes/no), and c) I would feel okay if people in my community knew (yes/no).

**Data Storage and Confidentiality:**

**Research Quality Assurance**

**RA training:** Training of RAs in the administration of assessment instruments will include: 1) A didactic teaching program by Dr. Chen on the recognition of signs and symptoms of depression in the elderly; 2) observation and subsequent discussion of training tapes; 3) observation of live interviews conducted by experienced clinicians using participants demonstrating a broad range of depression severity; 4) role-played interviews; and 5) supervised experience in conducting interviews with actual patients. We will follow the same procedures for RA training that has yielded reliable ratings in the Hangzhou study, which uses many of the same measures. Drs. Chen and Mao will provide ongoing supervision, with periodic review of the reliability of all clinical ratings. Finally, we will routinely check RA reliability in extracting data on utilization and cost.

**Data Management and Security*:*** Data obtained at subject assessments will be entered directly onto hardcopy forms by the RA. These data forms will be transferred monthly by the RC from the Tonglu data collection site to the Department of Psychology in ZJU in a locked briefcase. There the data will be checked and entered into the study’s central database for further cleaning, error checking, and secure, redundant storage according to procedures developed and overseen by ZJU and UR biostatisticians.

**Data Analysis and Monitoring:**

**Data Analysis Plan**

**Sample Size Calculation:** Primary outcomes of the Dep/HTN COACH Study are (1) adherence to depression and HTN treatment (MPR and % of subjects with 80% MPR, and MMAM); (2) depression symptom change (proportion with 50% reduction in HDRS and % with HDRS < 10); and (3) HTN control (% with BP controlled). (4) Improvements in quality of life outcomes (WHOQOL domain scores) and (5) cost differences associated with the interventions will be measured as well. We will assume an attrition rate of 20% over t12 months. Since the village-randomized study is a 3-level nested longitudinal design, power depends on the intra-class correlation (ICC) among the patients within the PCP and serial correlation between repeated assessments within the patient. We set the serial correlation at 0.5 as in most studies, but varied the ICC over 0.05, 0.1 and 0.2 to get a sense of the impact of the latter on power. Based on two-sided type I error = 0.05, power = 0.8 and an attrition rate of 20%, the detectable effect size ranged from 0.17 to 0.26 for a continuous outcome. Since experiences with multi-level designs suggest that ICC is generally smaller than 0.2, the study is sufficiently powered to detect small effect sizes between the two intervention groups. For the dichotomous outcomes, we also set the serial correlation at 0.5 and varied the percent of variance between patients within the PCP over 0.05, 0.1 and 0.2. Based on two-sided type I error = 0.05, power = 0.8, base rate 0.5 (most conservative) and an attrition rate of 20%, the detectable between-group proportion ranged from 11% to 17%, which is well within the range of clinically meaningful differences in the primary care setting.

**Data Analysis**

**General considerations:** All statistical tests are two-sided with p<.05. Descriptive statistics (counts and proportions for categorical variables and means [±SD] for continuous outcomes) will be used to depict the characteristics of the sample (e.g., age, gender, physical and functional status). We will compare baseline characteristics between groups using t-test (for continuous variables) and chi-square (for discrete variables), and examine associations between outcome variables and patients’ characteristics. Characteristics significantly differentiating the two groups, we will be treated as covariates when testing between-group differences using longitudinal models and structural equation models(SEM)(see below).

**Aims 1 and 2** will be addressed by generalized linear mixed effect models (GLMM) and weighted generalized estimating equations (WGEE), while **Aim 3** will be investigated by SEM. The primary outcomes are temporal changes of pertinent variables over the 12-month period, which we will model using longitudinal data methods based on the five assessments over 12 months. GLMM and WGEE are the two preferred approachesfor modeling treatment differences over time[120, 121]. Both accommodate continuous (e.g., MPR, HRDS) and binary responses (e.g., HDRS < 10) and provide valid inference in the presence of missing data if the missing value follows the missing at random (MAR) assumption, a popular mechanism that applies to most studies in practice [120, 121]. If estimates differ between the two approaches, only WGEE results will be reported, as it provides significantly more robust inference, especially in the presence of missing data [120-122]. We will perform intention-to-treat analyses using all subjects randomized to the treatment groups. All analyses will be conducted using SAS 9.2 or later.

For SEM, the maximum likelihood estimates (MLE) are also valid under MAR, if the posited distribution models are satisfied. In the presence of missing data, estimates from GLMM and SEM may be biased if the parametric assumptions are not met, even with the use of robust variance estimates [122]. If this issue arises, we will change the distribution models and/or use clustered bootstrap methods for inference [123]. Commonly used indices for assessing goodness of fit include likelihood ratio, Akaike and Bayesian criteria. For SEM, popular goodness-of-fit measurements include chi-square test, the comparative fit index, the index of Tucker and Lewis, and Root Mean Square Error of Approximation [124]. We will build models using an integrated strategy by combining forward selection, backward elimination, and goodness of fit procedures.

**Analysis Plan by Specific Aim**

**Aim 1 – Depression:** We hypothesize that relative to subjects who receive CAU, those in the COACH intervention will show (a) better adherence to antidepressant treatment recommendations and (b) greater improvements in their depressive disorders over 12 months of involvement in the study. We model each repeatedly assessed variable of adherence to depression treatment as well as improvement in depression using the longitudinal methods described above. For each outcome, time, intervention, and time by intervention interaction will be predictors, adjusting for the covariates identified above under general conditions. To avoid oversimplifying temporal patterns with linear trend, we will use piece-wise or even polynomial functions of time based on the assessment points. Linear contrasts will be used to assess COACH vs. CAU differences over the 12-month period as well as any sub time intervals within this period.

**Aim 2 – Hypertension:** We hypothesize that relative to subjects who receive CAU, those in the COACH intervention will show (a) better adherence to antihypertensive treatment recommendations and (b) greater improvements in BP control. The same approach as Aim 1 will be used to examine the hypotheses in this Aim.

**Aim 3 – Temporal associations:** We will examine the temporal associations of change in depression and BP control, hypothesizing that (a) improvements in treatment adherence precede improvement in depression and HTN, and (b) improvements in depression will precede improvements in BP control. The dynamic relationships between adherence, improvement in depression and BP control and mediation of the intervention effect on BP control by improvement in depression will be examined using SEM [125, 126]. We will first examine the causal, or meditational, relationship between COACH intervention, adherence to antidepressant (antihypertensive) medications and improvement in depression (BP control), using SEM with intervention as the predictor, adherence as the mediator and depression (BP control) as the outcome. To ensure the temporal order that changes in adherence take place before improvements in depression (BP control), the depression (BP control) variable will lag by one visit with respect to the adherence variable. Direct, indirect, and total effects will be constructed, and tests will be performed to see if adherence to antidepressant (antihypertensive) medications mediates the effect of the intervention on improvement in depression (BP control).

The same approach will be used to test the mediation hypothesis in (b), where depression is the mediator and BP control is the outcome.

**Aim 4 – Health-Related Quality of Life:** In addition to its impact on depression and BP control, we will ask whether the COACH intervention results in greater improvements in health-related quality of life than CAU. The same approach used in Aim 1 will be used to test the hypothesis. The longitudinal model will be applied to each of the WHOQOL domain scores.

**Aim 5 – Cost:** Finally, we will determine resource utilization and costs associated with delivering the CAU and the COACH interventions over 12 months and, in a set of *exploratory* analyses, conduct an economic feasibility evaluation using cost-effectiveness (CEA) and cost-benefit analysis methods (CBA). They consider them exploratory because the 12-month duration of the intervention is relatively short for such analyses.


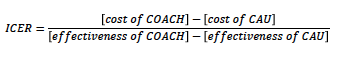
For CEA we will calculate an incremental cost-effectiveness ratio (ICER) based on two outcomes: (a) reduction in the average HDRS score between the CAU and COACH subjects (ΔEffect = H[CAU] – H[Coach]); and (b) Increase in the proportion of patients with controlled BP: ΔEffect = cBP(CAU) – cBP(Coach). The ICER will be interpreted as the incremental cost per unit improvement in health as measured by the outcome of choice (HDRS score or % of patients with BP control).

For CBA, the benefit of the intervention will be estimated as the reduction in the average healthcare expenses between the patients receiving CAU and COACH (ΔEffect =ΔBenefit = MedCost[CAU] – MedCost[Coach]). The results of cost-benefit analysis could be interpreted as the ROI (the ratio of reduction in medical costs to the incremental cost of the intervention, with ratio>1 indicating a significant return) or as a net monetary benefit (NMB=the difference between the reduction in medical costs and the incremental cost of the intervention, NMB>0 indicating a monetary benefit of the intervention).

**Data and Safety Monitoring Plan (DSMP)**

- 1. **Mission.** The purpose of the Data and Safety Monitoring Plan (DSMP) is to ensure the safety of participants and the validity and integrity of the data. It specifies who will look at the data and review any adverse events, how often, and what they are authorized to do. We will include a Data Safety Monitoring board (DSMB) as part of the monitoring plan as the best means of systematically reviewing the safety and integrity of the study.
  2. **DSMB Membership.** The DSMB will consist of three members based in China who are unconnected to the study and who have relevant expertise in each of three areas. They include the following:
- Chair: Xiao Shuiyuan, MD – overall familiarity with the health issues, conduct of clinical trials, and the Chinese cultural context in which the study is conducted. Prof. Xiao is Professor of Social Medicine &Psychiatry and Dean, School of Public Health, Central South University in Changsha, Hunan Province, China.
- Bioethicist: Li Lingjiang, Professor and Chair, Mental Health Institute, Central South University, Changsha, Hunan Province, China
- Biostatistician: Shen Mowei, Professor and Chair, Department of Psychology, Zhejiang University, Hangzhou, Zhejiang Province, China.
  1. **DSMB Responsibilities**
- Review and approve, disapprove, or suggest modifications to the study protocol and/or consent documents to assure both scientific integrity and that the study adheres to human subject protection policies.
- Monitor, provide feedback, and report on scientific and ethical issues related to study implementation for the protection of human subjects and advise on ethical issues related to adverse events. The DSMB will monitor adverse event reports for purposes of determining whether their nature, frequency and severity are consistent with expectations.
- Ensure that the study protocol maintains subjects’ confidentiality in a manner that is appropriately balanced with issues of clinical care and safety, where relevant.
- To monitor data management activities. The DSMB may ask to review data relevant to quality control. The DSMB will review requests for interim analyses and approve, disapprove, require additional information, or defer decisions.
  1. **Meeting Schedule and Data Reporting.** The DSMB will meet every 6 months by conference call, to include the three board members, Drs. Conwell and Chen, and the Hangzhou-based study coordinator. As well, the DSMB will be convened for conference calls whenever indicated in real time to address emergent concerns that may impact the study’s risk: benefit ratio or require reconsideration of the risk for participation as depicted in the study’s IRB-approved consent documents. These may include (1) an unexpected volume of expected serious adverse events, (2) a single event that is particularly severe or sensitive in nature (i.e., international scrutiny, or (3) any unexpected problems involving risks to subjects or others involved in the study.

The Board Chair will determine the format of the meetings. They will always include, for routine meetings, review of reports prepared and submitted by the China PI, Dr. Chen, one week prior to the scheduled meeting concerning subject recruitment and flow, any adverse events that occurred, and data quality assurance.

- 1. **Reporting of Adverse Events.** We will abide by the rules governing reporting of adverse events as defined in NIMH Policy on Data and Safety Monitoring in Clinical Trials (September 2002). Any event will be reported if it is either “serious,” “unexpected,” or “related,” or if it is of a “nature that may require international scrutiny.”
     1. **Reportable Events: Definition of terms**
        1. “**Serious**” means any event that causes a prolonged or permanent harm that is psychological, social, legal or financial. Examples most pertinent to the COACH study include a subject’s death from any cause; a suicide attempt or hospitalization due to depression, uncontrolled hypertension or its consequences (e.g., stroke).
        2. “**Unexpected**” means that the event was unforeseen and has not been previously encountered, known, or recognized and was not identified in nature, severity, or degree of incidence in the protocol, supporting documentation, the informed consent document, or the RSRB application.
        3. “**Related to the study**” means that there is some aspect of the study (e.g., a research procedure, existence of a laptop database, etc.) that is directly related to the event. An example pertinent to the COACH study is breach of confidentiality by which private information about the subject was made known to other community members. Events for which the relationship to the study can not be clearly determined based on review of all available data will be classified as “**possibly related to the study**” and reported according to the same guidelines as for related events.
        4. “Of a nature that may result in **international scrutiny**” refers to any event that could potentially have implications for relationships between the United States and China of which government agencies should be aware. Examples pertinent to the COACH study could include transfer of data deemed sensitive to U.S. or Zhejiang Provincial authorities without prior approval, or any adverse event that could take on a political rather than purely scientific dimension (e.g., by reporting in the press).
     2. **Procedures for recording and reporting AEs by study personnel to PI**

All potential AEs will be systematically recorded in a study log by the providers involved in the study – PCPs in CAU villages, and PCPs, AWs, and psychiatrists in COACH villages. The log utilized will include information identifying the subject, the type of event, the time and place it occurred, the harm or potential harm, and actions taken. It will include the definitions of AEs and SAEs, and guidelines specifying that they notify Dr. Chen by telephone *within 5 working days* of any SAE. Additionally, providers will also be instructed to report *immediately* to him any SAE that is judged to be related or possibly related to the study, and any that could potentially result in international scrutiny. Providers will be trained in use of the log at the outset of the study. Research coordinators will retrieve the logs on their quarterly visits to the villages. They will be maintained in locked files at the Tonglu Mental Hospital study site, from which they will be collected and transported to Zhejiang University by Research Coordinators during their monthly visits.

- - 1. **Procedures for recording and reporting AEs by PI to DSMB/IRB/study sponsors**

When Dr. Chen receives the logs, he will review them for three purposes: (1) to assure that no reportable SAEs were missed; (2) to designate for each event whether or not is was serious, unanticipated, and related to the study; (3) to identify any potential trends in events that become recognizable over time. He then will report AEs based on the following guidelines.

- *Unanticipated problems that are serious adverse events* will be reported to the DSMB Chair and both IRBs within 1 week of Dr. Chen becoming aware of the event.
- *Any other unanticipated problem* will be reported to the DSMB Chair and both IRBs within 2 weeks of Dr. Chen becoming aware of the problem.
- *All unanticipated problems* will also be reported to the NIMH Program Officer and to the Office of Human Research Protections within one month of the IRB’s receipt of the report of the problem from the investigator.
- *All AEs that are serious and either unexpected or related/possibly related* and suggest that the research may place subjects at greater risk of harm than previously known or recognized will be reported to the DSMB Chair and both IRBs immediately, and to the Office of Human Research Protections promptly thereafter.
- *All AEs that may result in international scrutiny* will be reported to the DSMB Chair, both IRBs, and the NIMH Program Officer as soon as possible, and within 3 business days.
- *All AEs that are serious but also expected and unrelated to the study* will be reported biannually to the DSMB and annually to the ZJU and UR IRBs and to NIMH as part of routine progress reports.

**IV. RISK/ BENEFIT ASSESSMENT**

**Risk Category:** This study poses minimal risk. UR researchers will only be managing and analyzing the data. The data will include identifiers but there are many protections in place to maintain the confidentiality of the subjects.

**Potential Risks:** As the study deals with a sample of older adults with both depression and HTN, risks that would be expected during its course include those that are related, or possibly related, to study participation and those that are solely associated with the natural course of either disease process or its treatment.

**Expected risks related to study participation** by older adult subjects include invasion of privacy, breach of confidentiality, or mild reactions to the research interviews of distress or fatigue. A risk particular to PCPs and AWs is that their participation could influence their performance evaluations, or impact on their employment, by CDC or CoA.

- **Expected risks unrelated to study participation** include those associated with the natural history of depression and of HTN, and any risks associated with their treatment.
  - **Depression and its treatment:** Risks associated with having depression are that the condition will worsen impairing functioning, weight loss or weight gain, insomnia or hypersomnia, self-harming behavior including suicide attempts and suicide, and the need for referral to a mental health specialist or hospitalization. Medication treatments for depression have a wide range of side effects as well that may include changes in blood pressure with a risk of falls, gastro-intestinal distress, and altered sexual functioning.
  - **HTN and its treatment:** Risks associated with having HTN include dizziness and headache, fatigue, blurred vision or blindness, worsening coronary heart disease, edema, strokes, and renal insufficiency or kidney failure. Medications commonly used for treatment of HTN can lead to low blood pressure and dizziness or falls, extra urination and altered sexual functioning, difficulty controlling blood sugar in diabetics, constipation, cough, swelling and rash.
  - Risks due to depression, HTN, and their treatments are present in the COACH and CAU arms of the study, as the treatments used are routine and available to PCPs either directly or through referral to a mental health professional in both study arms.

**Adequacy of protection against risks:**  Risks that could be related to participation in the study will be minimized by using only trained and clinically-experienced psychiatric nurses as research interviewers (RAs). In both COACH and CAU practices, PCPs will be informed of the PHQ-9 screening results and if evidence of suicide risk is revealed. All patients are under care of their PCPs who administer routine care, either as CAU or by standard treatment guidelines and care management. Treated patients are monitored regularly by the PCP for potential side effects, adverse reactions, and medication adherence. Moreover, practices of PCPs in the COACH arm will be supervised by a psychiatrist, while those in the CAU arm have routine access to psychiatric consultation by referral to the Tonglu County Mental Hospital. Consistent with routine practice, antidepressant medications will only be recommended when the patient’s regimen does not include drugs with which it is known to interact and when there is no other contraindication. If during baseline or any follow up assessment subjects are found to have conditions that place them at risks for adverse events, including suicidal ideation, or psychotic symptoms, the subject’s PCP will be notified immediately and referral to psychiatric care recommended.

For the protection of the confidentiality of OA, PCP, and AW subject information, a number of precautions will be taken, including training of research interviewers in confidentiality procedures; using coded identification labels for data entry and storage; restricted access by enforced password protection.

In order to help assure that their participation does not influence the PCPs’ and AWs’ employment status, data obtained from them will not be accessible to CDC and CoE personnel.

**Potential Benefits of the Proposed Research**

The potential benefits include:

- Early identification of depression (all OA subjects);
- Treatment of major depression based on current standards of care for a subset of patients who might otherwise not receive treatment (COACH intervention subjects),
- Improved medical outcomes for patients’ HTN treatment (COACH intervention subjects),
- Improved knowledge of the effect of an intervention on the identification and treatment of depression and HTN in village primary care patients, serving to guide subsequent service system design by Zhejiang CDC and Zhejiang CoA.
- Improved skills and knowledge by PCPs and AWs who receive training for the study.

With the precautions taken, the benefits to both individuals and society, in our view, greatly outweigh the risks of the study.

**V. SUBJECT IDETIFICATION, RECRUITMENT AND CONSENT/ASSENT**

**Sources of research material**

- **Older Adult subjects (OAs):** Two sources of data will be used. (1) Routine visits and in-person interviews of patients. Interviews will be conducted by the RA in the subject’s home, the PCP’s office, or the village aging association, depending on the subject’s preference. (2) Administrative data bases: Information on medical status and use of practice services will be extracted from the medical (clinic) record for each patient and from the Zhejiang Province health insurance system. The clinic record is an EMR record for each patient that documents his or her health history, vital signs, medications prescribed, and referrals made. The provincial insurance data base is linked to the individual subject’s EMR and records billing information for inpatient or outpatient medical care received at the township or county level, laboratory tests performed, and medications prescribed.
- **PCPs and AWs:** Data will be obtained at baseline and again after 1 and 2 years by interview with the RA and by questionnaires.

**Informed consent**

- **OAs:** Verbal informed consent will be obtained by the RA who will read the consent form to the subject, explaining that the intervention the subject receives (COACH or CAU) is determined by the prior random assignment of the clinic in which the subject receives his or her care. The RA will explain the study’s purpose and requirements for participation; the nature of the intervention they would receive (CAU or COACH) based on the village in which they reside; its risks and benefits; protections against risk and the subject’s rights. It will be made clear that their choice whether or not to participate will have no bearing on the care they receive from their PCP or the support they can expect from their village’s Aging Association. Patients will be told during the consent process that if he or she is found on assessment to be at imminent risk for harm to self or others, that his or her PCP will be informed. The RA will evaluate the subject’s capacity to provide fully informed consent by asking a series of questions to determine his or her level of understanding of the material presented. Subjects will be asked at that point if they have any questions or concerns, and if they agree to participate. Agreement will then be attested to by the RA who signs and dates the form, providing a copy to the subject for their own keeping.
- **PCPs & AWs:** PCPs and AWs will also be asked to provide verbal informed consent prior to study entry and after their village has been identified as a potential study site. All of the same essential elements will be included in the process.
- **Justification for verbal consent:** The choice to seek verbal rather than written consent was based, in consultation with and approval of the Zhejiang University IRB, on several factors. First is the literacy level of elderly rural Chinese older adults, many of whom have low levels of educational attainment and rudimentary reading skills. Second, village residents in this age group rarely sign contracts; doing so will raise their anxiety regardless of the content or purpose of the document, Third, obtaining and documenting verbal consent for research is the typical, culturally accepted practice in community settings, including for providers (e.g., village doctors). Dr. Chen’s ongoing study with urban Hangzhou older adults with depression has used this approach since its outset without concern raised by any participants. Discussions with the Zhejiang University IRB led to the conclusion that verbal consent was both appropriate to the cultural context and necessary in order to complete the study successfully.

**Costs to Subject:** There are no costs to subjects.

**Payment for Participation**: Older Adult subjects will receive $3.00(USD) for each visit. Payment will be made in Chinese currency(20 RMB).

PCPs (and AW-for COACH arm of the study) will be reimbursed for time used to conduct study procedure-10% of their salary and benefits.

**REFERENCES**

1. Ma X, Xiang YT, Li SR, Xiang YQ, Guo HL, Hou YZ, et al. Prevalence and sociodemographic correlates of depression in an elderly population living with family members in Beijing, China. PsycholMed. 2008;38(12):1723-30.

2. Zhang J, Ye M, Huang H, Li L, Yang A. Depression of chronic medical inpatients in China. Arch Psychiatr Nurs. 2008;22(1):39-49.

3. Blazer DG. Depression in late life: review and commentary. J Gerontol A Biol Sci Med Sci. 2003;58(3):249-65.

4. Chobanian AV, Bakris GL, Black HR, Cushman WC, Green LA, Izzo JL, Jr., et al. The Seventh Report of the Joint National Committee on Prevention, Detection, Evaluation, and Treatment of High Blood Pressure: the JNC 7 report. JAMA. 2003;289(19):2560-72.

5. Thom T, Haase N, Rosamond W, Howard VJ, Rumsfeld J, Manolio T, et al. Heart disease and stroke statistics--2006 update: a report from the American Heart Association Statistics Committee and Stroke Statistics Subcommittee. Circulation. 2006;113(6):e85-151.

6. MacKay J, Mensah G. The Atlas of Heart Disease and Stroke: World Heath Organization and Centers for Disease Control; 2004.

7. Lawes C, Hoorn S, Law M, Elliott P, MacMoham S, Rodgers A. High blood pressure. In: Ezzati M, Lopez AD, Rodgers A, Murray C, editors. *Comparative Quantification of Health Risks: Global and Regional Burden of Disease Attributable to Selected Major risk Factors*. Geneva, Switzerland: World Health Organization; 2004. p. 281-389.

8. Prince MJ, Ebrahim S, Acosta D, Ferri CP, Guerra M, Huang Y, et al. Hypertension prevalence, awareness, treatment and control among older people in Latin America, India and China: a 10/66 cross-sectional population-based survey. J Hypertens. 2012;30(1):177-87.

9. Davidson K, Jonas BS, Dixon KE, Markovitz JH. Do depression symptoms predict early hypertension incidence in young adults in the CARDIA study? Coronary Artery Risk Development in Young Adults. Arch Intern Med. 2000;160(10):1495-500.

10. Markovitz JH, Matthews KA, Kannel WB, Cobb JL, D'Agostino RB. Psychological predictors of hypertension in the Framingham Study. Is there tension in hypertension? JAMA. 1993;270(20):2439-43.

11. Meyer CM, Armenian HK, Eaton WW, Ford DE. Incident hypertension associated with depression in the Baltimore Epidemiologic Catchment area follow-up study. J Affect Disord. 2004;83(2-3):127-33.

12. Jonas BS, Franks P, Ingram DD. Are symptoms of anxiety and depression risk factors for hypertension? Longitudinal evidence from the National Health and Nutrition Examination Survey I Epidemiologic Follow-up Study. Arch Fam Med. 1997;6(1):43-9.

13. Kim MT, Han HR, Hill MN, Rose L, Roary M. Depression, substance use, adherence behaviors, and blood pressure in urban hypertensive black men. Ann Behav Med. 2003;26(1):24-31.

14. Morris AB, Li J, Kroenke K, Bruner-England TE, Young JM, Murray MD. Factors associated with drug adherence and blood pressure control in patients with hypertension. Pharmacotherapy. 2006;26(4):483-92.

15. Wang PS, Avorn J, Brookhart MA, Mogun H, Schneeweiss S, Fischer MA, et al. Effects of noncardiovascular comorbidities on antihypertensive use in elderly hypertensives. Hypertension. 2005;46(2):273-9.

16. Wang PS, Bohn RL, Knight E, Glynn RJ, Mogun H, Avorn J. Noncompliance with antihypertensive medications: the impact of depressive symptoms and psychosocial factors. J Gen Intern Med. 2002;17(7):504-11.

17. Prince M, Patel V, Saxena S, Maj M, Maselko J, Phillips MR, et al. No health without mental health. Lancet. 2007;370(9590):859-77.

18. Moussavi S, Chatterji S, Verdes E, Tandon A, Patel V, Ustun B. Depression, chronic diseases, and decrements in health: results from the World Health Surveys. Lancet. 2007;370(9590):851-8.

19. Murray CJ, Lopez AD. Alternative projections of mortality and disability by cause 1990-2020: Global Burden of Disease Study. Lancet. 1997;349(9064):1498-504.

20. WHO. *The Global Burden of Disease: 2004 Update*. Geneva, Switzerland: World Health Organization; 2008.

21. Smith SM, Allwright S, O'Dowd T. Effectiveness of shared care across the interface between primary and specialty care in chronic disease management. Cochrane Database Syst Rev. 2007(3):CD004910.

22. Chen R, Wei L, Hu Z, Qin X, Copeland JR, Hemingway H. Depression in older people in rural China. Arch Intern Med. 2005;165(17):2019-25.

23. Huang J. Recognition and intervention of depression symptoms in in-patients. Nursing Research (China). 205;10:295-6.

24. Zhang XC. *The urban-rural differences of inflation in China. 2009*; Available from: <http://www.bis.org/repofficepubl/arpresearch201003.16.pdf>. Accessed 6-10-12

25. Statistics ZBo. 2011 *Year Book Zhejiang*. 2011; Available from: <http://www.zj.stats.gov.cn/zjtj2011/indexch.htm>. Accessed 5-25-12

26. United Nations DoEaSA. *World Population Ageing 2009*. 2009.

27. Ministry of Health of People's Republic of China. *The National Guidelines for the Establishment of Primary Care Settings*. Beijing2009; Available from: <http://www.moh.gov.cn/publicfiles/business/htmlfiles/zwgkzt/pzcfg/index.htm>. Accessed 6-10-12

28. Chen S, Conwell Y, Xu B, Chiu H, Tu X, Ma Y. Depression care management for late-life depression in China primary care: protocol for a randomized controlled trial. Trials. 2011;12:121.

29. Phillips CO, Wright SM, Kern DE, Singa RM, Shepperd S, Rubin HR. Comprehensive discharge planning with postdischarge support for older patients with congestive heart failure: a meta-analysis. JAMA. 2004;291(11):1358-67.

30. Gilbody S, Bower P, Fletcher J, Richards D, Sutton AJ. Collaborative Care for Depression: A Cumulative Meta-analysis and Review of Longer-term Outcomes. Arch Intern Med. 2006;166(21):2314-21.

31. Bruce ML, Ten Have T, Reynolds CF, III, Katz IR, Schulberg HC, Mulsant BH, et al. Reducing Suicidal Ideation and Depressive Symptoms in Depressed Older Primary Care Patients: A Randomized Controlled Trial. JAMA. 2004;291(9):1081-91.

32. Unutzer J, Katon W, Callahan CM, Williams JW, Jr., Hunkeler E, Harpole L, et al. Collaborative care management of late-life depression in the primary care setting: a randomized controlled trial. JAMA. 2002;288(22):2836-45.

33. Wolff JL, Boult C. Moving beyond round pegs and square holes: restructuring Medicare to improve chronic care. Ann Intern Med. 2005;143(6):439-45.

34. Katon W, Lin EHB, Von Korff M, Ciechanowski P, Ludman E, Young B, et al. Integrating depression and chronic disease care among patients with diabetes and/or coronary heart disease: the design of the TEAMcare study. Contemp Clin Trials. 2010;31(4):312-22.

35. Chen S, Conwell Y, Van Orden K, Lu N, Fang U, Jin T, et al. Prevalence and natural course of late-life depression in China primary care: a population based study from an urban community. J Affect Disord:In press.

36. Spitzer RL, Williams JBW, Gibbon M. *Structured Clinical Interview for DSM-IV (SCID).* New York: New York State Psychiatric Institute, Biometrics Research; 1994.

37. Haynes RB, Taylor DW, Sackett DL. *Compliance in Health Care*. Baltimore: Johns Hopkins University Press; 1979.

38. Grymonpre RE, Mitenko PA, Sitar DS, Aoki FY, Montgomery PR. Drug-associated hospital admissions in older medical patients. Journal of the American Geriatrics Society. 1988;36(12):1092-8.

39. Hope CJ, Wu J, Tu W, Young J, Murray MD. Association of medication adherence, knowledge, and skills with emergency department visits by adults 50 years and older with congestive heart failure. Am J Health-System Pharmacy. 2004;61(19):2043-9.

40. Balkrishnan R, Rajagopalan R, Camacho FT, Huston SA, Murray FT, Anderson RT. Predictors of medication adherence and associated health care costs in an older population with type 2 diabetes mellitus: a longitudinal cohort study. Clin Therapeutics. 2003;25(11):2958-71.

41. Irvine J, Baker B, Smith J, Jandciu S, Paquette M, Cairns J, et al. Poor adherence to placebo or amiodarone therapy predicts mortality: results from the CAMIAT study. Canadian Amiodarone Myocardial Infarction Arrhythmia Trial. Psychosomatic Med. 1999;61(4):566-75.

42. Tedlow JR, Fava M, Uebelacker LA, Alpert JE, Nierenberg AA, Rosenbaum JF. Are study dropouts different from completers? Biol Psychiatry. 1996;40(7):668-70.

43. Melfi CA, Chawla AJ, Croghan TW, Hanna MP, Kennedy S, Sredl K. The effects of adherence to antidepressant treatment guidelines on relapse and recurrence of depression. Arch Gen Psychiatry. 1998;55(12):1128-32.

44. Croghan TW, Lair TJ, Engelhart L, Crown WE, Copley-Merriman C, Melfi CA, et al. Effect of antidepressant therapy on health care utilization and costs in primary care. Psychiatr Serv. 1997;48(11):1420-6.

45. Bogner HR, Lin JY, Morales KH. Patterns of early adherence to the antidepressant citalopram among older primary care patients: the prospect study. Int J Psychiatry Med. 2006;36(1):103-19.

46. Organization WH. *Adherence to Long-Term Therapies: Evidence for Action*. Geneva, Switzerland: 2003.

47. Ryan AA. Medication compliance and older people: a review of the literature. Int J Nurs Stud. 1999;36(2):153-62.

48. Park DC, Morrell RW, Frieske D, Blackburn AB, Birchmore D. Cognitive factors and the use of over-the-counter medication organizers by arthritis patients. Hum Factors. 1991;33(1):57-67.

49. Young P, Dewse M, Fergusson W, Kolbe J. Respiratory rehabilitation in chronic obstructive pulmonary disease: predictors of nonadherence. Eur Respir J. 1999;13(4):855-9.

50. Peyrot M, McMurry JF, Jr., Kruger DF. A biopsychosocial model of glycemic control in diabetes: stress, coping and regimen adherence. J Health Soc Behav. 1999;40(2):141-58.

51. Garay-Sevilla ME, Nava LE, Malacara JM, Huerta R, Diaz de Leon J, Mena A, et al. Adherence to treatment and social support in patients with non-insulin dependent diabetes mellitus. J Diabetes Complications. 1995;9(2):81-6.

52. Demyttenaere K. Compliance during treatment with antidepressants. J Affect Disord. 1997;43(1):27-39.

53. Wetherell JL, Unutzer J. Adherence to treatment for geriatric depression and anxiety. CNS Spectr. 2003;8(12 Suppl 3):48-59.

54. Keller MB, Hirschfeld RM, Demyttenaere K, Baldwin DS. Optimizing outcomes in depression: focus on antidepressant compliance. Int Clin Psychopharmacol. 2002;17(6):265-71.

55. Callahan CM, Hendrie HC, Tierney WM. The recognition and treatment of late-life depression: a view from primary care. International J Psychiatry Med. 1996;26(2):155-71.

56. Meredith LS, Rubenstein LV, Rost K, Ford DE, Gordon N, Nutting P, et al. Treating depression in staff-model versus network-model managed care organizations. J Gen Intern Med. 1999;14(1):39-48.

57. Rost K, Nutting P, Smith J, Coyne JC, Cooper-Patrick L, Rubenstein L. The role of competing demands in the treatment provided primary care patients with major depression. Arch Fam Med. 2000;9(2):150-4.

58. Cooper LA, Gonzales JJ, Gallo JJ, Rost KM, Meredith LS, Rubenstein LV, et al. The acceptability of treatment for depression among African-American, Hispanic, and white primary care patients. Med Care. 2003;41(4):479-89.

59. Conwell Y, Lyness JM, Duberstein P, Cox C, Seidlitz L, DiGiorgio A, et al. Completed suicide among older patients in primary care practices: a controlled study. J Am Geriatr Soc. 2000;48(1):23-9.

60. Conwell Y, Duberstein PR, Cox C, Herrmann JH, Forbes NT, Caine ED. Relationships of age and axis I diagnoses in victims of completed suicide: a psychological autopsy study. Am J Psychiatry. 1996;153(8):1001-8.

61. Duberstein PR, Conwell Y, Conner KR, Eberly S, Caine ED. Suicide at 50 years of age and older: perceived physical illness, family discord and financial strain. PsycholMed. 2004;34(1):137-46.

62. Duberstein PR, Conwell Y, Conner KR, Eberly S, Evinger JS, Caine ED. Poor social integration and suicide: fact or artifact? A case-control study. PsycholMed. 2004;34(7):1331-7.

63. Tsoh J, Chiu HF, Duberstein PR, Chan SS, Chi I, Yip PS, et al. Attempted suicide in elderly Chinese persons: a multi-group, controlled study 2. Am J GeriatrPsychiatry. 2005;13(7):562-71.

64. Yip PSF, Chi I, Chiu H, Wai KC, Conwell Y, Caine E. A prevalence study of suicide ideation among older adults in Hong Kong SAR. Int J Geriatr Psychiatry. 2003;18(11):1056-62.

65. Chan SM, Chiu FK, Lam CW, Leung PY, Conwell Y. Elderly suicide and the 2003 SARS epidemic in Hong Kong. Int J Geriatr Psychiatry. 2006;21(2):113-118.

66. Holt-Lunstad J, Smith TB, Layton JB. Social relationships and mortality risk: A meta-analytic review. PLoS Med. 2010;7(7):e1000316.

67. De Leo D, Dello Buono M, Dwyer J. Suicide among the elderly: the long-term impact of a telephone support and assessment intervention in northern Italy. Br J Psychiatry. 2002;181:226-9.

68. Motto JA, Bostrom AG. A randomized controlled trial of postcrisis suicide prevention. PsychiatrServ. 2001;52(6):828-33.

69. Oyama H, Watanabe N, Ono Y, Sakashita T, Takenoshita Y, Taguchi M, et al. Community-based suicide prevention through group activity for the elderly successfully reduced the high suicide rate for females. Psychiatry Clin Neurosci. 2005;59(3):337-44.

70. Morrow-Howell N, Becker-Kemppainen S, Judy L. Evaluating an intervention for the elderly at increased risk of suicide. Res Social Work Prac. 1998;8:28-46.

71. Richardson TM, Friedman B, Podgorski C, Knox K, Fisher S, He H, et al. Depression and Its Correlates Among Older Adults Accessing Aging Services. J Nurs Care Qual. 2011.

72. Richardson TM, Simning A, He H, Conwell Y. Anxiety and its correlates among older adults accessing aging services. Int J Geriatr Psychiatry. 2011;26:31-8.

73. Simning A, Richardson TM, Friedman B, Boyle LL, Podgorski C, Conwell Y. Mental distress and service utilization among help-seeking, community-dwelling older adults. Int Psychogeriatr. 2010;22(5):739-49.

74. Chen S, Chiu H, Xu B, Ma Y, Jin T, Wu M, et al. Reliability and validity of the PHQ-9 for screening late-life depression in Chinese primary care. Int J Geriatr Psychiatry. 2010;25(11):1127-33.

75. Kroenke K, Spitzer RL, Williams JB. The PHQ-9: validity of a brief depression severity measure. J Gen Intern Med. 2001;16(9):606-13.

76. Liu, L-S, Writing Group of 2010 Chinese Guidelines for the Management of Hypertension. *Chinese guidelines for the management of hypertension*. Beijing 2010.

77. Callahan CM, Unverzagt FW, Hui SL, Perkins AJ, Hendrie HC. Six-item screener to identify cognitive impairment among potential subjects for clinical research. Med Care. 2002;40(9):771-81.

78. Sheehan DV, Lecrubier Y, Sheehan KH, Amorim P, Janavs J, Weiller E, et al. The Mini-International Neuropsychiatric Interview (M.I.N.I.): the development and validation of a structured diagnostic psychiatric interview for DSM-IV and ICD-10. J Clin Psychiatry. 1998;59 Suppl 20:22-33.

79. Si TM, Shu L, Dang WM, Su YA, Chen JX, Dong WT, et al. Evaluatin of teh reliability and validity of a Chinese version of the Mini-International Neuropsychiatric Interview in patients with mental disorders. Chinese Ment Health J. 2009;23:493-7.

80. Krahn DD, Bartels SJ, Coakley E, Oslin DW, Chen H, McIntyre J, et al. PRISM-E: comparison of integrated care and enhanced specialty referral models in depression outcomes. PsychiatrServ. 2006;57(7):946-53.

81. Schulberg HC, Block MR, Madonia MJ, Scott CP, Lave JR, Rodriguez E, et al. The 'usual care' of major depression in primary care practice. ArchFamMed. 1997;6(4):334-9.

82. Katon W, von KM, Lin E, Bush T, Ormel J. Adequacy and duration of antidepressant treatment in primary care. MedCare. 1992;30(1):67-76.

83. Lin EH, Von KM, Katon W, Bush T, Simon GE, Walker E, et al. The role of the primary care physician in patients' adherence to antidepressant therapy. MedCare. 1995;33(1):67-74.

84. Callahan CM, Dittus RS, Tierney WM. Primary care physicians' medical decision making for late-life depression. JGenInternMed. 1996;11(4):218-25.

85. Wagner EH, Austin BT, Davis C, Hindmarsh M, Schaefer J, Bonomi A. Improving chronic illness care: translating evidence into action. Health Aff. 2001;20(6):64-78.

86. Wagner EH, Simon GE. Managing depression in primary care. BMJ. 2001;322(7289):746-7.

87. Katon W, Unutzer J. Collaborative care models for depression: time to move from evidence to practice. ArchInternMed. 2006;166(21):2304-6.

88. Katon W, Von KM, Lin E, Simon G. Rethinking practitioner roles in chronic illness: the specialist, primary care physician, and the practice nurse. GenHospPsychiatry. 2001;23(3):138-44.

89. Pro-Change Behavior Systems I. *Roadways to Healthy Living: A Guide for Managing High Blood Pressure*. Pro-Change Behavior Systems I, editor. West Kingston, RI: Pro-Change Behavior Systems, Inc.; 2009.

90. Pro-Change Behavior Systems I. *Roadways to Healthy Living: A Guide for Depression Prevention.* Pro-Change Behavior Systems I, editor. West Kingston, RI: Pro-Change Behavior Systems, Inc.; 2009.

91. Steffens DC, McQuoid DR, Krishnan KR. The Duke Somatic Treatment Algorithm for Geriatric Depression (STAGED) approach. PsychopharmacolBull. 2002;36(2):58-68.

92. Han C, Voils CI, Williams JW, Jr. Uptake of Web-Based Clinical Resources from the MacArthur Initiative on Depression and Primary Care. Community Ment Health J. 2011.

93. Sheehan DV, Lecrubier Y, Sheehan KH, Amorim P, Janavs J, Weiller E, et al. The Mini-International Neuropsychiatric Interview (M.I.N.I.): the development and validation of a structured diagnostic psychiatric interview for DSM-IV and ICD-10. J Clin Psychiatry. 1998;20:22-33.

94. Morisky DE, Ang A, Krousel-Wood M, Ward HJ. Predictive validity of a medication adherence measure in an outpatient setting. J Clin Hypertens. 2008;10(5):348-54.

95. Xu W-H, Wang Q, Liang W-X. Reliability and validity of Morisky Questionnaire in measurement of the compliance with hypertension medication (Chinese). Chinese J Prevention Control of Chronic Non-communicable Diseases. 2010;15(5):424-6.

96. Wong MC, Jiang JY, Griffiths SM. Antihypertensive drug adherence among 6408 Chinese patients on angiotensin-converting enzyme inhibitors in Hong Kong: a cohort study. J Clin Pharmacol. 2010;50(5):598-605.

97. Halpern MT, Khan ZM, Schmier JK, Burnier M, Caro JJ, Cramer J, et al. Recommendations for evaluating compliance and persistence with hypertension therapy using retrospective data. Hypertension. 2006;47(6):1039-48.

98. Mallion JM, Baguet JP, Siche JP, Tremel F, de Gaudemaris R. Compliance, electronic monitoring and antihypertensive drugs. J Hypertens Suppl. 1998;16(1):S75-9.

99. Zheng YP, Zhao JP, Phillips M, Liu JB, Cai MF, Sun SQ, et al. Validity and reliability of the Chinese Hamilton Depression Rating Scale. BrJ Psychiatry. 1988;152:660-4.

100. Lecrubier Y. How do you define remission? Acta Psychiatrica Scandinavica, Suppl. 2002;415:7-11.

101. Bogner HR, Cary M, Bruce ML, Reynolds III CF, Mulsant BH, Ten Have TR, et al. The role of medical comorbidity in outcome of major depression in primary care: the PROSPECT study. Am J Geriatr Psychiatry. 2005;13(10):861-8.

102. Organization WH. Development of the World Health Organization WHOQOL-BREF quality of life assessment. The WHOQOL Group. Psychol Med. 1998;28(3):551-8.

103. Skevington SM, Lotfy M, O'Connell KA, Group W. The World Health Organization's WHOQOL-BREF quality of life assessment: psychometric properties and results of the international field trial. A report from the WHOQOL group. Qual Life Res. 2004;13(2):299-310.

104. Leung KF, Wong WW, Tay MS, Chu MM, Ng SS. Development and validation of the interview version of the Hong Kong Chinese WHOQOL-BREF. Quality of Life Research. 2005;14(5):1413-9.

105. Xia P, Li N, Hau KT, Liu C, Lu Y. Quality of life of Chinese urban community residents: a psychometric study of the mainland Chinese version of the WHOQOL-BREF. BMC Med Res Methodol. 2012;12:37.

106. Yang SC, Kuo PW, Wang JD, Lin MI, Su S. Development and psychometric properties of the dialysis module of the WHOQOL-BREF Taiwan version. J Formosan Med Assoc. 2006;105(4):299-309.

107. Attkisson CC, Zwick R. The client satisfaction questionnaire. Psychometric properties and correlations with service utilization and psychotherapy outcome. EvalProgram Plann. 1982;5(3):233-7.

108. Larsen DL, Attkisson CC, Hargreaves WA, Nguyen TD. Assessment of client/patient satisfaction: development of a general scale. Eval Program Plann. 1979;2(3):197-207.

109. Charlson ME, Pompei P, Ales KL, MacKenzie CR. A new method of classifying prognostic comorbidity in longitudinal studies: development and validation. J Chronic Dis. 1987;40(5):373-83.

110. Chu YT, Ng YY, Wu SC. Comparison of different comorbidity measures for use with administrative data in predicting short- and long-term mortality. BMC Health Serv Res. 2010;10:140.

111. Tong AY, Man DW. The validation of the Hong Kong Chinese version of the Lawton instrumental activities of daily living scale for institutionalized elderly persons. Occupational Therapy Journal of Research: Occupation, Participation and Health. 2002;22:132-42.

112. Chen MR, Guo QH, Cao XY, Hong Z, Liu XH. A preliminary study of the Six-Item Screener in detecting cognitive impairment. Neurosci Bull. 2010;26(4):317-21.

113. Yu DS, Lee DT, Woo J. Psychometric testing of the Chinese version of the medical outcomes study social support survey (MOS-SSS-C). Res Nurs Health. 2004;27(2):135-43.

114. Ruan D, Linton CF, Dai X, Pan Y, Zhang W. On the changing structure of social networks in urban China. Soc Networks. 1997;19:75-89.

115. Uher R, Farmer A, Henigsberg N, Rietschel M, Mors O, Maier W, et al. Adverse reactions to antidepressants. Br J Psychiatry. 2009;195(3):202-10.

116. Testa MA, Hollenberg NK, Anderson RB, Williams GH. Assessment of quality of life by patient and spouse during antihypertensive therapy with atenolol and nifedipine gastrointestinal therapeutic system. Am J Hypertens. 1991;4(4 Pt 1):363-73.

117. Keller MB. Undertreatment of major depression. Psychopharmacol Bull. 1988;24(1):75-80.

118. Alexopoulos GS, Meyers BS, Young RC, Kakuma T, Feder M, Einhorn A, et al. Recovery in geriatric depression. Arch Gen Psychiatry. 1996;53(4):305-12.

119. Givens JL, Katz IR, Bellamy S, Holmes WC. Stigma and the acceptability of depression treatments among african americans and whites. J Gen Intern Med. 2007;22(9):1292-7.

120. Kowalski JT, X.M. *Modern Applied U Statistics*. New York: Willey; 2007.

121. Tang WH, H.; Tu, X.M. Applied Categorical and Count Data Analysis. Chapman & Hall/CRC; Florida2012.

122. Lu N, Tang W, He H, Yu Q, Crits-Christoph P, Zhang H, et al. On the impact of parametric assumptions and robust alternatives for longitudinal data analysis. Biometrical Journal Biometrische Zeitschrift. 2009;51(4):627-43.

123. Field CAW, A.H. Bootstrapping clustered data. J Royal Statistical Soc: Series B (Statistical Methodology). 2007;69:12.

124. Conner KR, Gunzler D, Tang W, Tu XM, Maisto SA. Test of a clinical model of drinking and suicidal risk. Alcoholism Clin Experiment Res. 2011;35(1):60-8.

125. Cole DA, Maxwell SE. Testing mediational models with longitudinal data: questions and tips in the use of structural equation modeling. J Abnormal Psychol. 2003;112(4):558-77.

126. Tu XM, Feng C, Kowalski J, Tang W, Wang H, Wan C, et al. Correlation analysis for longitudinal data: applications to HIV and psychosocial research. Statistics in Medicine. 2007;26(22):4116-38.
