# Supplementary material for: Effectiveness of integrated care for older adults with depression and hypertension in rural China: A cluster randomized controlled trial
Source: PLoS Med. 2022 Oct 24;19(10):e1004019. doi: 10.1371/journal.pmed.1004019 (PMC9639850; doi:10.1371/journal.pmed.1004019)
Supplement: Attachment — Submitted filename: Responses to reviewers 10-2-22.pdf [file pmed.1004019.s011.pdf]

RESPONSES TO REVIEWERS' AND EDITORS' COMMENTS

Title: Effectiveness of integrated care for older adults with depression and hypertension in rural China: a cluster randomized controlled trial

PMEDICINE-D-22-01526R2

Thank you again for your comments on the paper and the opportunity to respond to them with this revised version. In this document we present our response to each of the Editor's comments and to those of Reviewer #4. The other reviewers had no remaining concerns.

Comments are reproduced in *italics* followed by our responses in regular type. We provide with each response an explanation and the location (page and line numbers) of where one can find corresponding changes that resulted. (Note that these locations refer to the "clean" version of the revised text.)

Comments from the **EDITOR**:

#1. *Lines 59-60: Please do not report P=0.0001; report as P < 0.001.*

Response: We have made that change.

#2. *Abstract: Please define the following abbreviations on first use: HTN, PHQ, PCP, OR, LMIC (abbreviations are only necessary if the term is used more than once).*

Response: We have made changes to the first mention of each of these abbreviations to include their full definitions in the Abstract, pages 2-4. We also checked the main text to assure that the same conventions were used there as well.

#3. *Author Summary: Please include the headline numbers from the study, such as the sample size and key findings.*

Response: In the revised Author Summary on page 5, lines 89-91 we added the sample size information. Based on review of other published papers in the journal, we refer to the key findings descriptively rather than by including the numerical statistics because doing so seemed more technical than intended for this section. We would be glad to add more details of that nature if you prefer.

#4. *Please update your TIDieR checklist to use section and paragraph numbers rather than page numbers.*

Response: We have updated the TIDieR checklist so that it now refers to section and paragraph numbers. The checklist is included as S3 Appendix.

#5. *Line 342: Please change text colour to black.*

Response: We apologize that there were numerous bits of the manuscript where a red font rather than black was erroneously used. We have corrected each of those instances.

#6. *Table 2: Please consider presenting the crude (unadjusted) analysis as Supporting Information*.

Response: We have added results of unadjusted analyses for the primary outcomes as S6 Table, and refer the reader to it on page 20, line 371.

#7. *Line 393: S1 Table does not contain information regarding demographic and clinical characteristics at baseline, please check whether this citation is incorrect.*

Response: The reference to S1 Table in the prior version was an error. We have removed that citation.

#8. Line 526: Please remove 'however'.

Response: We have made that change.

Comments from **REVIEWER #4**

#1. *Being a trial, I think you need a subheading in the methods describing the main outcomes.*

Response: We appreciate the suggestion and have added a subheading in the Methods section labeled "Primary Outcomes" on page 13, line 273.

#2. *Related to that point, I would encourage the authors/editors to maintain uniformity in the entire document, including text, table and figures about the sequence of how the outcomes are presented (e.g. depression goes always first and hypertension second).*

Response: Again, thank you for noticing this inconsistency. Throughout the manuscript we now mention depression before HTN, and present results and discussion points concerning each in that order. The most substantive change in that regard is found in the discussion from page 27, line 464 through page 29, line 505, where we now address observations concerning the large effect sizes for depression before discussing those for HTN.
