# Supplementary material for: Effectiveness of integrated care for older adults with depression and hypertension in rural China: A cluster randomized controlled trial
Source: PLoS Med. 2022 Oct 24;19(10):e1004019. doi: 10.1371/journal.pmed.1004019 (PMC9639850; doi:10.1371/journal.pmed.1004019)
Supplement: Attachment — Submitted filename: Responses to reviewers 8-22-22.docx [file pmed.1004019.s010.docx]

**RESPONSES TO REVIEWERS’ AND EDITORS’ COMMENTS**

Title: Effectiveness of integrated care for older adults with depression and hypertension in rural China: a cluster randomized controlled trial

PMEDICINE-D-22-01526R1

Thank you all very much for your comments on the paper and the opportunity to respond to them with this revised version. We found the comments helpful and trust that our careful consideration of them, and our responses to them, have resulted in an improved product.

In this document we present our response to each of the Academic Editor’s and four reviewers’ comments in sequence. Comments are reproduced in *italics* followed by our responses in regular type. We provide with each response an explanation and the location (page and line numbers) of where one can find corresponding changes that resulted. (Note that these locations refer to the “clean” version of the revised text.) In those instances where we provide an excerpt from the revised manuscript, we distinguish the text by indenting and placing it in “quotes”.

Comments from the **ACADEMIC EDITOR**:

#1. *Please provide more comment on the exceptionally large effect size from the HTN treatment arm. There was an approximate three-fold increase in control rates in the intervention arm. This is really out of keeping with effect sizes seen in most implementation research trials. It would be helpful if the authors could provide an explanation for this in more detail.*

**Response:** Thank you for pointing out the need for greater explanation of the large effect size in HTN control in the COACH arm. It is likely to reflect several factors. We have added the following wording to the Discussion section to address these possibilities on pg 27-28:

“The almost three-fold increase in HTN control rates in COACH intervention recipients could be accounted for by several factors. First, HTN control may have increased with improvement in depressive symptoms due to increases in the hypertensive subject’s health behaviors, including activity, diet, and social interactions. The post hoc comparison of those COACH participants who did and did not agree to take an antidepressant showed no difference in the proportions that achieved HTN control, suggesting that the medication was not the driver of improvement. More likely is that the AW, who was charged with supporting participants’ adherence to medication and behavioral treatments for HTN, may have positively influenced the outcome as planned. It is also possible, however, that more frequent and more systematic follow-up by AWs and PCPs in the COACH group than in eCAU created more opportunities to detect uncontrolled BP, resulting in more active hypertension management. Finally, the older patients and their PCPs were aware that HTN control was a subject of study and may have increased adherence to treatment for that reason. The same explanation may explain the two-fold increase in HTN control found in the eCAU group as well. Unfortunately, we do not have access to information on rates of adherence to HTN treatment guidelines by participants in either COACH or eCAU groups with which to examine these possibilities further.”

#2. *Please provide a more detailed description of intervention components, using the TIDieR Checklist.*

**Response:** We have included the TIDieR Checklist (S3 Appendix) with this submission referencing where in the text to find the additional details that we now provide. As well, we have added a table as Supporting Information (S1 Table) that describes the roles and responsibilities of each member of the COACH team.

#3. *Please report on antihypertensive medication use by randomized group. Please also report reduction in blood pressure as a continuous measure. It would be helpful to comment on whether the large effect size over 12 months is related to use of BP medication, or any interventions that were taken to enhance BP medication use.*

**Response:** As we now explain on pg 9, lines 167-169, all individuals with a diagnosis of HTN in the medical record are prescribed antihypertensive medications according to guidelines established by the China Center for Disease Control and Prevention. All participants in both study arms, therefore, received antihypertensive prescriptions. Unfortunately, we do not have information available on their actual use of these medications. This limitation is acknowledged in the Discussion, pg 28, lines 478-480. We have data on BP readings from which the outcome of HTN control was calculated, as explained on pg 11, lines 202-205. In addition to the findings described in the text, we have now calculated the mean systolic and diastolic BP readings at each assessment point for COACH and eCAU participants. As they were not specified outcomes, we include that information now as Supporting Information (S2 Table).

With regard to potential explanations of the large effect size, please refer to our response to your first concern (item #1).

#4. *Please report the ICC for primary outcomes (please see CONSORT for Cluster Trials item 17a).*

**Response:** We apologize for this oversight. The ICCs for the primary outcomes are now included on pg 20-21, 377-379 and line 384-385.

*Other editorial points:*

#5. *Data availability statement: Thank you for your willingness to make your data available in the Harvard Dataverse. Please provide the DOI/accession number*.

**Response:** The COACH study is now published on the Harvard Dataverse, including the study’s code book and raw data file. Please refer to <https://doi.org/10.7910/DVN/6RWH41>

#6. *Throughout: Please use “participants” rather than “subjects” when describing the individuals who participated in the trial*.

**Response:** As suggested, we have replaced “subject” with “participant” throughout the manuscript.

#7. *Abstract: Please include the trial registration information in the abstract*.

**Response:** We now include the ClinicalTrials.gov identifier in the Abstract (pg. 2, lines 30-31).

#8. *Abstract: Methods and Findings: Please provide the specific start and end dates of the trial.*

**Response:** We have modified the Abstract to include the following information on pg 2, lines 30-32:

“The study, registered with ClinicalTrials.gov as Identifier NCT01938963, was a cluster randomized controlled trial with 12-month follow-up conducted from January 1, 2014 through September 30, 2018, with analyses conducted in 2020-2021.”

#9. *Abstract: Methods and Findings: Line 41: Please mention how “controlled HTN” was determined.*

**Response:** We have added the following description to the Abstract, pg 3, lines 48-51:

“…the proportion with controlled HTN, defined as systolic BP <130 mmHg or diastolic BP <80 for participants with diabetes mellitus, coronary heart disease or renal disease, and systolic BP <140 or diastolic BP <90 for all others..”

#10. *Abstract: Methods and Findings: Please specify who was blinded to the intervention and control.*

**Response:** We have modified the Abstract to include the following information on pg 3:

“Observers, older adult participants, and their PCPs were blinded to study hypotheses but not to group assignment.”

#11. *Abstract: Please state that analysis was intention to treat. Please provide the number of participants lost to follow up in each group.*

**Response:** We have modified the Abstract to include the following information on pg 3, lines 51-57:

“Analyses were conducted using generalized linear mixed effect models with intention to treat …. Sixty-seven of 1133 participants assigned to eCAU and 85 of 1232 COACH participants were lost to follow-up over 12 months. They included 36 participants who died of natural causes, 22 in the COACH arm and 14 receiving eCAU. Forty COACH participants discontinued antidepressant medication due to side effects.”

#12. *Abstract: Methods and Findings: Please quantify the main results with both 95% CIs and p values*.

**Response:** We have added p values to the 95% CI for the effect sizes in the Abstract on pg 3, lines 58-60.

#13. *Abstract: Methods and Findings: Please provide some summary baseline data/description of the participants*.

**Response:** We have added the following summary description of the participants to the Abstract on pg 2.

“Of 2899 eligible village residents, 2365 (82%) agreed to participate. They had a mean age of 74.5 years, 67% were women, 55% had received no schooling, 59% were married and 20% lived alone.”

#14. *Abstract: Methods and Findings: As mentioned by a reviewer, please focus the abstract on the presentation of primary and secondary pre-specified outcomes, rather than exploratory analyses.*

**Response:** We have removed the portion concerning results of exploratory analyses from the last line of the Abstract’s Methods and Findings section.

#15. *Abstract: Methods and Findings: Please include a summary of adverse events if these were assessed in the study*.

**Response:** We have added the following to the Abstract, pg 3.

“They included 36 participants who died of natural causes, 22 in the COACH arm and 14 receiving eCAU. Forty COACH participants discontinued antidepressant medication due to side effects.”

#16. *Abstract: Methods and Findings: In the last sentence of the Abstract Methods and Findings section, please describe the main limitation(s) of the study's methodology.*

**Response:** The following text has been added to the Abstract on pg 3, lines 62-66:

“Limitations of the study’s methodology include the inability to mask research assessors and participants to which condition a village was assigned, and lack of information about participants’ adherence to recommendations for lifestyle and medication management of HTN and depression. Generalizability of the model to other regions of China or other LMICs may be limited.”

#17. *Abstract: Conclusions: Please address the study implications without overreaching what can be concluded from the data; the phrase "In this study, we observed ..." may be useful.*

**Response:** Thank you for pointing out the need to restrict conclusions to those that can be drawn from the findings of the study. We have replaced that section of the Abstract (pgs 3-4, lines 68-71 with the following:

“Conclusions: In this study we observed that the COACH model of integrated care management resulted in greater improvement in both HTN control and depression symptom severity among older adult residents of rural Chinese villages who had both conditions than did eCAU.”

#18. *Author summary: At this stage, we ask that you include a short, non-technical Author Summary of your research to make findings accessible to a wide audience that includes both scientists and non-scientists. The Author Summary should immediately follow the Abstract in your revised manuscript. This text is subject to editorial change and should be distinct from the scientific abstract. Please see our author guidelines for more information*: <https://urldefense.proofpoint.com/v2/url?u=https-3A__journals.plos.org_plosmedicine_s_revising-2Dyour-2Dmanuscript-23loc-2Dauthor-2Dsummary&d=DwIGaQ&c=4sF48jRmVAe_CH-k9mXYXEGfSnM3bY53YSKuLUQRxhA&r=G7hwJfzTVxTr4i9VvsKENbpZUpSzjrhWjhIoVUA3Vn1gs8sUIY2Ck_ulKC0EvBuh&m=J6DK48tzrrzOBLcV_Q1iS031n2B84FnUhLK4Mb93A60&s=wn7yDR49gaVPt-dv1M3_Vz1kAitgcvM7YVVe_fp_AHQ&e=>

**Response:** We have added the Author Summary following the Abstract on pgs 5-6.

#19. *Introduction: Line 69: Here and throughout the manuscript, please refer to high income countries rather than "developed" or "Western" countries.*

**Response:** Rather than use other terms, we now refer to high-income countries throughout the manuscript.

#20. *Methods: Protocol: Please include the study protocol document and analysis plan, with any amendments, as Supporting Information to be published with the manuscript if accepted. Please highlight all changes between what was done and what was prespecified in the protocol.*

**Response:** The study protocol, including the analysis plan, is included as Supporting Information (S1 Appendix). We have highlighted in the document any changes between what was done and what was prespecified in the protocol, and provided explanations in the margins for those changes.

#21. *Methods: Please ensure that the study is reported according to the CONSORT guideline. Please add the following statement, or similar, to the Methods: "This study is reported as per the Consolidated Standards of Reporting Trials (CONSORT) guideline (S1 Checklist)."*

*Thank you for including the completed CONSORT checklist as Supporting Information. When completing the checklist, please use section and paragraph numbers (e.g. Methods, paragraph 1), rather than page numbers*.

**Response:** We have included the completed CONSORT and TIDieR checklists as Supporting Information (S2 Appendix and S3 Appendix) using the section and paragraph numbers as recommended. As well, we have added the following wording to the Methods section, pg. 16:

“The study is reported as per the Consolidated Standards of Reporting Trials (CONSORT) Extension to Cluster Randomised Trials [43] (see Supporting Information S2 Appendix) and the Template for Intervention Description and Replication (TIDieR) checklist (S3 Appendix).”

#22. *Methods: Line 127: Please describe the sociodemographic information collected at baseline. Please describe how social network size was determined*.

**Response:** Specific information about the sociodemographic information collected at baseline is now included in the Methods section, where we also have added description of the measurement of social network size, pg 10, lines 181-190:

“Sociodemographic information collected at baseline included participants’ age, sex, marital status and living situation (alone, with spouse and/or children, or with others), years of education, employment status, religion (Buddhist, Christian, Islam, none), and level of economic satisfaction (very sufficient to very poor). … Social network size was assessed with the Lubben Social Network Scale (LSNS), a continuous measure of perceived social support received from family and friends [31].”

#23. *Methods: Line 135: Please provide some brief background on the HDRS measure of depressive symptoms, including scoring.*

**Response:** On page 10, lines 193-197 we have added the following background for the HDRS and its scoring:

“The 17-item HDRS assesses symptoms of depressive illness including mood, anxiety, neurovegetative symptoms of energy, appetite and sleep, anhedonia, psychomotor change and somatic concerns. Each item is scored on a three- or five-point scale with total score representing their sum (range, 0-52). Higher scores indicate greater depressive symptom severity.”

#24. *Methods: line 184-185: Please explicitly specify all primary and secondary outcome measures of the study. The following outcomes measures appear to differ between the submitted manuscript and the trial registry: The registered primary outcome is Depressive Symptom Change and HTN control is not mentioned, except as an Other Outcome Measure. Health related quality of life at each timepoint is not described. Costs associated with intervention are not described. Assessment of outcomes at time points prior to the 12 month assessment are also not described. Please clarify and explain the discrepancy. If the outcomes were not prespecified in the protocol, please indicate that they were post hoc and explain why they were added. Post hoc comparisons should be presented as hypothesis generating rather than conclusive.*

**Response:** The primary outcomes of the study were always intended to be change in depression symptom severity (HDRS total score) and the proportions (%) of participants whose HTN was controlled, as explained on pg 10-11, lines 194 and 201. We apologize for any discrepancy with the description in ClinicalTrials.gov entry. As indicated in the study protocol, included in the submission as Supporting Information (S1 Appendix), the first three aims of the study were to determine whether COACH is more effective than CAU in treating depression (Aim 1) and HTN (Aim 2), and to examine the temporal relationships between treatment adherence and depression and HTN outcomes. I am sorry that we cannot reconstruct exactly how we mistakenly listed HTN as an Other Outcome Measure rather than primary in the trial registry. Health related quality of life is described in this submission as measured by the WHOQOL-BREF scale (see pg 10, line 187. However, we omitted the words “health-related” in its description. We have made that correction:

“Other measures assessed health-related quality of life using the WHOQOL-BREF [27,28], …”

Adherence to treatment and cost metrics are listed as outcome measures in the study protocol and trials registry as well, but are not included in this paper. Just before beginning data collection, the measure we proposed to use for adherence was withdrawn from public use by its creator who was unwilling to provide an exemption for research. We hoped instead to construct an adherence measure from analyses of medication prescription data, along with cost calculations from that and related administrative sources. Doing so proved very difficult given the large number of patients and clinics involved, and the complexity of Chinese governmental policies and procedures. To this point we have been unable to obtain a complete data set on adherence and cost. Rather than further delay publication of the findings reported here, we decided to address them in a separate manuscript at a later date.

The only *post hoc* variable included here is the one designed to categorize COACH participants as accepting an antidepressant prescription (Antidep[+]) or not (Antidep[-]). It was created after the study was completed in order to explore the role of medication use in depression and HTN control outcomes (see pg 16, lines 323), where they are described as “*post hoc*” and their use as “exploratory”.

#25. *Methods: Line 243-244: Please define "lost to follow-up" as used in this study. Other reasons for exclusion should be defined*.

**Response:** We have expanded description of inclusion and exclusion criteria in the Participants subsection on pg 8-9, lines 164-177. As well, we have now added the definition of “lost to follow-up” on pg 16.

“Reasons for lost-to-follow up, categorized as deceased, unable to contact, and refused further assessment, are depicted for COACH and eCAU groups in Fig 1, the study’s CONSORT diagram.”

#26. *Results: Please present numerators and denominators for percentages, at least in the Tables [not necessarily each time they're mentioned].*

**Response:** We have provided numerators and denominators for percentages reported in Tables 1 and 2. In Table 1, to avoid redundancy, we do so only for the first row of figures in for each variable, since the remaining rows will be the same. For example, on pg. 16\ we provide the numerator and denominator only for the percentage of men in the total group and then the eCAU and COACH subgroups. For the women we provide only the numerator since the denominator remains no different. We did not provide numerators and denominators in the text, only % figures, since the details are available in the tables themselves. We are glad to display the figures differently if you prefer another approach.

#27. *Results: Line 255-263: Please quantify results presented in the text, 95% CIs and p values for depression scores and HTN outcomes, as well as group x time interactions*.

**Response:** We have added the 95% CIs and *p* values along with the effect sizes and ICCs to the text on pg 20, lines 385-387.

#28. *Results: Line 266-275: Please quantify the comparison between those who did and did not accept prescribed antidepressants, in terms of differences in HTN control and HDRS scores*.

**Response:** In the Results section we have added findings of the comparison of those who did (Antidep[+]) and those who did not accept prescribed antidepressants (Antidep[-]) with regard to HTN control and HDRS scores. As well, we added a column providing those comparisons to S1 Table. The language added to the text can be found on pg 24, lines 391-403:

“Comparing those who did accept antidepressants (Antidep[+]) with those who did not (Antidep[-]), there were no notable differences in demographic and clinical characteristics at baseline (S1 Table). Only HDRS score distinguished the two groups (Antidep[+] (SD) = 22.6 (4.8); Antidep[-] (SD) = 21.4 (4.0); *p*<0.001), although the absolute difference of 1.2 HDRS points is unlikely to be clinically meaningful. Figs 2 and 3 allow for visual comparison of eCAU with Antidep[+] and Antidep[-] subgroups. The COACH Antidep[-] group had a pattern of response in HDRS total score between that of eCAU and Antidep[+] (Fig 2). With regard to HTN control, a different pattern emerged. There was no meaningful difference between the COACH subgroups in the proportions that achieved HTN control (Antidep[+] = 27% vs.; Antidep[-] = ; *p*=0.11), but improvements in HTN control were clearly greater for both COACH subgroups than for eCAU participants as depicted in Fig 3.”

#29. *Discussion: Please present and organize the Discussion as follows: a short, clear summary of the article's findings; what the study adds to existing research and where and why the results may differ from previous research; strengths and limitations of the study; implications and next steps for research, clinical practice, and/or public policy; one-paragraph conclusion*.

**Response:** We have revised the discussion to follow this organizational structure.

#30. *Figure 2 and Figure 3: Please also present these data in Table format.*

**Response:** The data for Figures 2 and 3 are provided as Supporting Information in S4 Table and S5 Table respectively.

#31. *Figure 2: Please show the axis beginning at zero. If this is not possible, please show a break in the axis*.

**Response:** We have revised Figure 2 so that the y-axis begins at zero.

**REVIEWER #1: Statistical review**

*This paper reports a cluster randomised trial evaluating an intervention aimed at improving mental health and hypertension in over 60s in Chinese rural settings. The intervention significant improved both outcomes.*

*The trial is well reported and used appropriate statistical methods. I only had some minor comments to make.*

#1. *Abstract, methods and findings "study was a 12-month cluster randomized controlled" - I would move the 12-month part to the end of the sentence and reword as 'with 12 month follow-up'. Currently it reads strangely that the trial was 12 months but took place between 2014 and 2017.*

**Response:** Thank you for the suggestion. We have made those changes on pg 2, line 31 and page 8, line 136.

#2. *Abstract line 43: I would add p-values to the estimated effect sizes and CIs, as the trial was prospectively powered to test hypotheses. I do not think it is good practice, according to CONSORT, to report non-pre-specified analyses in the abstract (it's fine to report in results), but will leave that to the editor.*

**Response:** We have added p values to the 95% CI for the effect sizes in the Abstract on pg 3, lines: 58-60. Please refer as well to our responses to the editor’s queries, items #4, #12, and #27.

#3. *Discussion: was there a reason for strong differences in baseline between randomised groups? Presumably this is just due to the small number of clusters? Generally it is not advised to test for difference in baseline variables in RCTs but I would imagine the reasons for this would not apply to CRTs with small number of clusters.*

**Response:** Thank you for your thoughts on the observed differences between the COACH and eCAU groups in some variables at baseline. Although rural villages in the townships of Jiande and Tonglu Counties are of similar size, they may differ with regard to their economic (eg, proximity to industrial sites, availability of farming and non-farming jobs) and social characteristics (eg, histories of out-migration of younger people to urban center for work.) Because the number of clusters in the randomization of townships was relatively small, we felt it was indicated to control for the observed differences in subsequent analyses.

**REVIEWER #2**

*This is a welcome paper in the realm of physical and mental health multimorbidity from an LMIC and shows gratifying improvements in blood pressure control with the multifaceted COACH intervention. There are a few points that need clarification*

**ABSTRACT**

#1. *It is not clear how villages can be subjects as it stated*
#2. *Subjects were rural village clinics of randomly selected towns in Zhejiang Province, China. Please clarify how villages can be part of towns- this should be added to the methods in order to provide greater context for the reader.*

**Response:** Thank you for pointing out our awkward explanation of the sampling. Because it is a problem reflected in both comments #1 and #2, we respond to them together.

In China, provinces such as Zhejiang Province, are made up of towns, each of which in turn contains approximately 20 villages. Each village has on primary care clinic staffed by one doctor. In order to minimize the likelihood of contamination between study arms (PCPs sharing their experience with the COACH intervention with other colleagues from a village clinic assigned to deliver eCAU), we elected to randomize towns rather than villages, clinics, or individual participants. To help clarify the sampling and randomization, we now use the term “township” rather than “town” because the intended meaning may be clearer to readers, and revised the abstract as follows (pg 2, lines 33-38):

“Participants were residents of 218 rural villages in ten randomly selected townships of Zhejiang Province, China. Each village hosts one primary care clinic that serves all residents. Ten townships, which of which contains approximately 20 villages, were randomly selected, with all villages and their associated clinics in each township, to deliver either the Chinese Older Adult Collaborations in Health (COACH) intervention or enhanced care-as-usual (eCAU) to eligible village clinic patients. The COACH intervention consisted of …”

#3. *If the study was a 12-month cluster randomized controlled trial conducted from 2014 through 2017, when were the follow up assessments made and why were the analyses only conducted in 2020-2021?*

**Response:** Subject recruitment ended in 2017, but follow-up data collection continued until September 2018 (see Academic Editor, item #8). Due to the complexity of the study, large number of subjects and data collection sites, the next year was dedicated to management and cleaning/checking of the data set. Coinciding with completion of that process in December 2019, the corona virus was first recognized in Wuhan. The pandemic and response then slowed our progress in analysis and writing.

**METHODS**

#4. *How were the participants identified - was it on the day of attendance or from registries?*

**Response:** Potentially eligible participants were initially identified by PCP review of the clinic’s patient roles and subsequent screening for inclusion and exclusion criteria by the PCP and study personnel. The procedures for identifying and assessing potential participants are now more fully explained in revised text on pgs 8-9, lines 163-177.

#5. *It is not clear what the frequency of visits in the enhanced usual care was- was it the same as for the intervention group?*

**Response:** PCPs in eCAU clinics did not maintain activity logs. Rather, the frequency their visits with participants in usual care was determined by the China Center for Disease Control and Prevention guidelines for primary care management of HTN, which specifies a visit to the clinic every three months. We have added wording to the Discussion (pg 27, lines 471-473) acknowledging the possibility that this difference could explain the observed differences between the groups in HTN control. Please see the Academic Editor’s first comment (item #1) in this regard as well.

#6. *How generalisable would it be for psychiatrists to be available for primary care clinic visits in rural China outside cities?*

**Response:** Currently access to mental health care is poor in all areas of rural China. Acknowledging that there is more work to be done for the COACH model to be scalable in rural areas, we have added the following wording to the Discussion on pg 29, line 526:

“While the COACH intervention appears effective as administered, access to mental health care in rural areas remains limited. Further study is needed of modifications to COACH that may further increase its scalability, such as employment of telehealth technology to link rural village-based COACH teams to psychiatric supports remotely ….”

#7. *What is the explanation for the marked increase in blood pressure control in the EAU group?*

**Response:** This is an important issue, and raised by the Academic Editor with regard to the COACH group also (Editor item #1). Please see our response there, which included addition of the following wording to the Discussion, pg 27, line 473-476:

“Finally, the older patients and their PCPs were aware that BP control was a subject of study and may have increased adherence to treatment for that reason. The same explanation may explain the two-fold increase in HTN control found the the eCAU group as well.”

#8. *It is unfortunate that there were so many baseline differences between the 2 groups but the appropriate analyses were undertaken to account for these.*

**Response:** Thank you for the endorsement of our approach to this issue.

**DISCUSSION**

*In the discussion two other RCTs are referred to that have  examined collaborative care management of comorbid medical and mental disorders in LMIC- the one for depression and diabetes and the other depression and hypertension. The first demonstrated greater improvement in depression and DM control but no benefit was found in the latter.*

#9. *It would be helpful for the authors to tease out the components of the collaborative care in these three trials in an attempt to better understand why there were these differences. For example what role did the psychiatrists play in the current trial- one would assume a major one given the lack of difference in outcome in people who were on antidepressants compared to those who did not accept them.*

**Response:** As requested by Reviewer #4, we now reference a third prior study of treatment for comorbid depression and medical illness in LMICs on pg 26 (Araya and colleagues). We have added there a more complete review of the elements of the intervention used in each. Please refer to pgs 25-26, lines 425-460. In addition, we have further clarified the role of the psychiatrist in the COACH model on pg 12, lines 254-256 and 270, and S1 Table.

#10. *Some more detail of how the AWs supported the intervention group would be helpful.*

**Response:** The AW’s role is likely to have been especially important for HTN control, as explained now on pg 26, lines 469-471. We have added description of their backgrounds on pg 11, lines 223-226.

“Each village, in addition to one PCP, has one AW employed part-time by the village leaders. The great majority are women with a middle-school education who receive in-service training from the Bureau of Civil Affairs in addressing the villagers’ social needs. The COACH intervention linked the PCP and AW of each village remotely by telephone with one psychiatrist based at the County Mental Hospital.”

Their roles and responsibilities are now listed in S1 Table as well as the text (pg 11-12, lines 244-252:

“The AWs’ role was to educate participants about their conditions followed by goal setting and collaborative development of behavior change strategies. Their training curriculum included an overview of depression, HTN, and their relationship; principles of disease self-management; psychosocial assessment and care planning; psychoeducation with older adults and their families; and ethical standards including confidentiality. They did not receive training in manualized psychosocial interventions. During monthly home visits, AWs helped the older person to set behavioral goals, review progress, and modify the plan if needed. In addition, the AWs created social opportunities for older persons, for example, by organizing social activities and educational workshops on a monthly basis.”

Information about the frequency of their meetings with participants, PCPs, and the consulting psychiatrist is now provided on pg 19, lines 361-368 as well as S3 Table.

**REVIEWER #3**

*The authors should be congratulated for conducting and reporting on a remarkable trial of an integrated intervention for late-life depression and hypertension in rural China. This is the single largest study of its kind and it has multiple strengths including the large sample size (both in terms of the number of clinics and the number of patients enrolled) and also the high rate of enrollment and follow-up among eligible subjects. The intervention and the training efforts seem 'realistic' and should not be too challenging to replicate in other 'real world' settings. I do have a few suggestions for the final version of this manuscript.*

#1. *It is unfortunate that it was not possible to blind the assessors to intervention status. This could create rater biases that are hard to control for, especially since one of the main outcome measures (HDRS) is not a patient rating but an assessor-rated scale*.

**Response:** We agree, and acknowledge this limitation on pg 28, lines 507-509. We attempted to minimize the potential for biased assessments by blinding the research staff and participants to the study’s hypotheses, but recognize the limits of this approach.

#2. *The relative effectiveness of the intervention seems to be much greater for hypertension control than for depression. Can the authors comment on this in the discussion? The base rate of controlled hypertension was remarkably low and the effectiveness of the intervention on achieving control of hypertension was remarkably high - so from a public health perspective, this seems to be the primary benefit of this intervention. It is not clear from the paper if and how the focus on depression helped with hypertension control or what was done specifically in the intervention to achieve hypertension control. It is also not clear if a similar algorithm driven intervention focusing on blood pressure control could have achieved similar results for hypertension control. It would be helpful to have more information on changes and differences in the use of antihypertensive medications. It may also be possible that more frequent and more systematic follow-up simply created more opportunities for PCPs and their staff to detect the fact that hypertension was not (yet) controlled, resulting in more active hypertension management.*

**Response:** Thanks for raising this important set of issues. Because the reported results of HDRS effect size were a bit surprising (ie, a small effect size given the estimated between group difference of almost 7 HDRS points), we checked all of our analyses. We found an error in estimating the standard deviation of HDRS reduction and therefore the overall effect size. It should be -1.43 [-1.71, -1.15] rather than -0.21 [-0.25, -0.17]. All other results were confirmed. The revised figures are now reflected accurately in Fig 2 (pg 24), and references to the HDRS effect size have been corrected in the text (pg 3, line 59; pg 20, line 384).

We have tried to address the large effect of the intervention on HTN control more fully in the discussion. Please refer to our response to the Academic Editor item #1, and to the revised text on pg 27, lines 471-473 where we include this reviewer’s useful suggestion that the pronounced impact on HTN control could be due to more frequent and systematic tracking of BP by PCPs, independent from any effect of depression symptom improvement.

#3. *The effects on depression are significant but moderate. Several things may contribute to this. First, the benefits of antidepressant medications may be relatively greater for more severe forms of depression - and given that subjects were included who had a PHQ-9 screen of 10 or greater, there may be a number of patients with milder forms of depression who did not have much relative benefit from medications. Secondly, as the authors point out, nearly half of participants did not agree to take antidepressants. It might be helpful to augment the intervention with some relatively simple psychosocial intervention such as behavioral activation strategies that can be effective alternatives to medication or augmentation strategies and that can be provided by non-specialists.*

**Response:** These are all excellent points that we have attempted to address in the Discussion (pg 28, lines 495-497). There we address the interesting differences in both HTN control and depression symptom severity outcomes in those who did and did not accept an antidepressant, which suggests that optimizing acceptance of and adherence to the medication will have great benefit. We also have added the recommendation that further research be conduct on the addition of a psychosocial intervention to the model on pg 28, beginning at line 505:

“For the substantial minority of COACH subjects who are not willing to accept an antidepressant, future research is warranted on incorporation of easily adopted, evidence-based psychosocial intervention like behavioral activation that can be provided by non-specialists.”

#4. *Could the authors comment on baseline differences in depression severity and the WHOQOL-BREF. What might contribute to these differences and how where they addressed?*

**Response:** As noted in response to Reviewer1, item #3, there were several differences at baseline likely accounted for by a combination of factors. One, the fact that villages, although similar in size, differ to some extent in their socioeconomic characteristics, including factors that in turn impact health-related quality of life and the likelihood of developing depressive symptoms (eg, proximity to industrial sites, availability of farming and non-farming jobs, household income; degree of out-migration of younger and healthy people to urban centers for work.) Second, the randomization was based at the township level in order to reduce the risk of bias due to contamination of the intervention across village/clinic boundaries; therefore, the number of clusters to be accounted for in analysis was relatively small. We addressed these imbalances by entering the variables that distinguished the groups at baseline as covariates in analyses.

#5. *One minor comment: Page 10, line 208 - we cannot say for certain that there was a change in 'depressive disorders' but rather what was observed was a change in the severity of depression symptoms as assessed by the HDRS.*

**Response:** Thank you for pointing out our error, which we have now corrected on pg 14, line 300.

**REVIEWER #4**

*This is a relevant article targeting rural village clinics in Zhejiang Province, China, to deliver the Chinese Older Adult Collaborations in Health (COACH) intervention or enhanced care-as-usual (eCAU). The COACH intervention consisted of algorithm-driven treatment of depression and HTN by village primary care doctors supported by village lay workers with consultation from centrally-located psychiatrists. The primary outcomes of the study were change in depression symptom severity as measured by the HDRS at baseline and follow-up assessments, and the proportions (%) of subjects whose HTN was controlled.*

#1. *Introduction, lines 71-73, refers to a previous study in China by the same authors (PMID: 26360086). Could you expand what is the difference with the current study and why this study was needed?*

**Response:** Thank you for identifying the need for greater clarity on this point. Our prior study differed from COACH in several ways. First, it was conducted in urban clinics where both the training and access to resources of PCPs is greater, and where only a minority (about 25%) of Chinese people live; 75% live in rural precincts. Second, the prior study examined depression only, not a comorbid condition. Third, there was no social component to the integrated care of older people in the first study; the AW was added to the COACH intervention in order to help meet social needs that are common in the rural population. We have added an explanation of these differences on pg 27, lines 485-488:

“That prior study, which did not examine the impact of the collaborative care intervention on comorbid medical illnesses, included no rural residents and no AW or other lay team member was involved. It did, however, did use the same antidepressant medication algorithm as in COACH [22].”

#2. *Introduction, lines 71-72, the statement "Rarely, however, have studies been conducted in LMICs that used a randomized control trial (RCT) design" misses the most recent evidence arising from Latin America (see PMID: 33974019), which could complement this study. Again, this reference will also be uniquely suited to enhance the discussion provided in lines 290-297*.

**Response:** Thank you for pointing out the Araya et al article, which is an important complement to our study. As noted in our response to Reviewer 2, item #9, we have included a reference to it and added the article to an expanded discussion of the three interventions’ characteristics and findings.

#3. *Statistical analysis, lines 190-201, the authors present a detectable between-group proportion (for HTN control) indicating that calculated values of 11% to 17% are "well within the range of clinically meaningful differences in primary care settings". Yet, we this reviewer would like to see a similar remark for the depression outcomes, since the authors only report that "detectable effect size ranged from 0.17 to 0.26 for the continuous outcome of depression symptom change" and the reader does not know if such calculations imply clinically meaningful differences.*

**Response:** We have added the following wording to the Methods section (pg 13, lines 285-287) to provide this information, and added a reference:

“A 3 point between group difference, considered the minimal clinically important difference in HDRS score, corresponds to an effect size of 0.5. Therefore, our study was amply powered for that outcome [38].”

#4. *Methods, an ethical procedures section is missing. The information is scattered in other sections.*

**Response:** Thank you for pointing out this omission. We have now included a sub-section of Methods labelled “Ethical procedures” on pg 15.

#5. *Table 1 does not require p-values, as p-value is a measure for inferential purposes, not descriptive ones (PMID: 30096356).*

**Response:** Reviewer 1(item #3) also noted that, “*Generally it is not advised to test for difference in baseline variables in RCTs”,* but added*, “I would imagine the reasons for this would not apply to CRTs with small number of clusters.”* Because the number of clusters in the randomization of townships was relatively small, we felt it was indicated to control for the observed differences in subsequent analyses. We carefully considered the alternatives and, because we used the variables that differed as covariates in the analyses, felt it was appropriate to include the p-values in Table 1. We would be glad to reconsider the issue.

#6. *Results lacks a description of the delivery of the intervention, how many aging workers (AW), what was the turnover, how many replacements were needed, was re-training needed, was village-level or town-level tailoring required, how often did consultations with psychiatrists occur, etc., etc. This is a complex intervention that requires aligning to the TIDieR checklist (PMID: 24609605)*

**Response:** Thank you for pointing out the need for these details of the intervention. In addition to completing the TIDieR checklist as Supporting Information (S3 Appendix), we included a table listing PCP, AW, and Psychiatrist roles and responsibilities (S4Table), and have made these modifications to the Methods section of the text to explain the intervention more fully. From pg 10-11, lines 221-236:

“The COACH intervention team in each village consisted of the clinic’s PCP, the village AW, and a county hospital Psychiatrist. The background and qualifications of the PCP are as described above for eCAU. Each village, in addition to one PCP, has one AW employed part-time by the village leaders. The great majority are women with a middle-school education who receive in-service education from the Bureau of Civil Affairs in addressing the villagers’ social needs. The COACH intervention linked the PCP and AW of each village with one psychiatrist based at the County Mental Hospital. There was no turn-over in PCPs, AWs, or Psychiatrists during any village’s 12-month participation in the study and, because the staffing of health and social services does not differ between villages in the counties involved, there was no need for tailoring of the intervention between them.

“Recruitment of villages and participants within them took place over four waves. Training for each wave took place over four days immediately preceding the start of the intervention by that group. It included separate groups for instruction in the roles of the PCP, AW, and Psychiatrist and joint training in how to work together in delivering the intervention collaboratively and with fidelity.”

*Minor comments*

#7. *Abstract, lines 31-32, please clarify "Subjects were rural village clinics of randomly selected towns in Zhejiang Province, China."*

**Response:** We appreciate the awkwardness of the wording, and have revised the abstract description of the subjects and sampling. Please see Reviewer 2, item #2 above for further explanation, and the revised text on pg 2, lines 34-38.

#8. *Line 237 reads "The study protocol was prospectively registered with Grants.gov [41]", yet the correct Website is* [https://urldefense.proofpoint.com/v2/url?u=https-3A__clinicaltrials.gov_&d=DwIGaQ&c=4sF48jRmVAe_CH-k9mXYXEGfSnM3bY53YSKuLUQRxhA&r=G7hwJfzTVxTr4i9VvsKENbpZUpSzjrhWjhIoVUA3Vn1gs8sUIY2Ck_ulKC0EvBuh&m=J6DK48tzrrzOBLcV_Q1iS031n2B84FnUhLK4Mb93A60&s=LG6zZtKYP62sA38_f4V4k88NsdREPIkEMZryrFO16m8&e=](https://urldefense.proofpoint.com/v2/url?u=https-3A__clinicaltrials.gov_&d=DwIGaQ&c=4sF48jRmVAe_CH-k9mXYXEGfSnM3bY53YSKuLUQRxhA&r=G7hwJfzTVxTr4i9VvsKENbpZUpSzjrhWjhIoVUA3Vn1gs8sUIY2Ck_ulKC0EvBuh&m=J6DK48tzrrzOBLcV_Q1iS031n2B84FnUhLK4Mb93A60&s=LG6zZtKYP62sA38_f4V4k88NsdREPIkEMZryrFO16m8&e=  )

**Response:** We apologize for the error. Intended to direct the reader to the protocol listed in the ClinicalTrials.gov registry, the sentence should read as follows (pg 15, lines 330-331). The reference is correct.

“The study protocol was prospectively registered with Clinicaltrials.gov as Identifier NCT01938963 [42] and …”

*Discretionary comments*

#9. *Abstract, line 53: "geriatric mental health care is lacking", do you mean minimal or absent?*

**Response:** Thank you for pointing out the misstatement. It would have been more appropriate to say “minimal”. However, based on the editor’s feedback on the content of the Abstract’s conclusion (see Academic Editor, item #17), we have removed that sentence.
